# The E3 Ligase TRIM4 Facilitates SET Ubiquitin‐Mediated Degradation to Enhance ER‐*α* Action in Breast Cancer

**DOI:** 10.1002/advs.202201701

**Published:** 2022-07-17

**Authors:** Dianwen Han, Lijuan Wang, Li Long, Peng Su, Dan Luo, Hanwen Zhang, Zheng Li, Bing Chen, Wenjing Zhao, Ning Zhang, Xiaolong Wang, Yiran Liang, Yaming Li, Guohong Hu, Qifeng Yang

**Affiliations:** ^1^ Department of Breast Surgery, General Surgery Qilu Hospital of Shandong University Jinan Shandong 250012 China; ^2^ Pathology Tissue Bank Qilu Hospital of Shandong University Jinan Shandong 250012 China; ^3^ Mianyang Central Hospital School of Medicine University of Electronic Science and Technology of China Mianyang Sichuan 621000 China; ^4^ Department of Pathology Qilu Hospital of Shandong University Jinan Shandong 250012 China; ^5^ The Key Laboratory of Stem Cell Biology Institute of Health Sciences Shanghai Institutes for Biological Sciences Chinese Academy of Sciences & Shanghai Jiao Tong University School of Medicine University of Chinese Academy of Sciences Shanghai 200233 China; ^6^ Research Institute of Breast Cancer Shandong University Jinan Shandong 250012 China

**Keywords:** breast cancer, ER‐*α*, SET, tamoxifen, TRIM4, ubiquitination

## Abstract

Estrogen receptor alpha (ER‐*α*) action is critical for hormone‐dependent breast cancer, and ER‐*α* dysregulation can lead to the emergence of resistance to endocrine therapy. Here, it is found that TRIM4 is downregulated in tamoxifen (TAM)‐resistant breast cancer cells, while the loss of TRIM4 is associated with an unfavorable prognosis. In vitro and in vivo experiments confirm that TRIM4 increased ER‐*α* expression and the sensitivity of breast cancer cells to TAM. Mechanistically, TRIM4 is found to target SET, and TRIM4‐SET interactions are mediated by the RING and B‐box domains of TRIM4 and the carboxyl terminus of SET. Moreover, it is determined that TRIM4 catalyzed the K48‐linked polyubiquitination of SET (K150 and K172), promoting its proteasomal degradation and disassociation from p53 and PP2A. Once released, p53 and PP2A are able to further promote *ESR1* gene transcription and enhance mRNA stability. Moreover, univariate and multivariate Cox proportional hazards regression analyses confirm that TRIM4 expression is an independent predictor of overall survival and recurrence‐free survival outcomes in patients with ER‐*α* positive breast cancer. Taken together, the data highlights a previously undiscovered mechanism and suggest that TRIM4 is a valuable biomarker that can be analyzed to predict response to endocrine therapy in breast cancer patients.

## Graphical abstract

TRIM4 catalyzes the K48‐linked polyubiquitination of SET, promoting its proteasomal degradation and disassociation from p53 and PP2A. Once released, p53 and PP2A can further promote ESR1 gene transcription and enhance mRNA stability. The findings outline a promising therapeutic target for the remediation of TAM resistance in breast cancer and other diseases associated with aberrant SET expression.

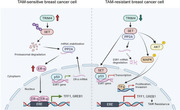

## 1 Introduction

Breast cancer remains the most prevalent form of cancer among women and is a leading cause of global morbidity and mortality.^[^ ^1^ ^]^ Approximately 70% of breast cancer patients present with estrogen receptor (ER)‐*α* positive disease, with such ER‐*α* expression being associated with better prognostic outcomes and a greater likelihood that patients will achieve therapeutic benefit from endocrine therapies including tamoxifen (TAM).^[^ ^2^ ^]^ Indeed, ER‐*α* expression level is a key determinant of TAM responsivity, and the deletion of ER‐*α* leads to an estrogen‐independent phenotype and TAM resistance in human breast cancer.^[^ ^3^ ^]^

It has been reported that ER‐*α* expression in human breast tumors is maintained by transcriptional or post‐transcriptional regulatory mechanisms. For example, the transcription of the *ESR1* gene, which encodes ER‐*α*, is controlled by p53 binding to its proximal promoter in concert with other transcription factors.^[^ ^4^ ^]^ Moreover, protein phosphatase 2A (PP2A) can influence the stability of the *ESR1* mRNA to shape the downstream expression of this hormone receptor.^[^ ^5^ ^]^ However, the mechanisms regulating ER‐*α* expression are not completely understood.

Previous studies have suggested that the SE translocation (SET) oncoprotein, which is also referred to as Template Activating Factor‐1*β* (TAF‐1*β*), is a prognostic biomarker that is associated with failed TAM treatment outcomes owing to its ability to modulate ER‐*α* signaling.^[^ ^6^ ^]^ As an endogenous inhibitor of the tumor suppressor PP2A, SET increases the tumorigenic potential of breast cancer cells.^[^ ^7^ ^]^ In addition, SET can suppress the transactivation of p53 through its ability to inhibit the p300/CBP‐dependent H3K18 and H3K27 acetylation at p53 target promoters,^[^ ^8^ ^]^ implying that SET may regulate the expression of ER‐*α* through the inhibition of PP2A and p53. However, the specific molecular mechanisms associated with these processes remain to be elucidated.

SET was shown to localize to the endoplasmic reticulum, cytoplasm, and nucleus wherein it can modulate cellular proliferation, apoptosis, and differentiation by altering the activity of the MAPK, Ra1A, c‐Myc, and AKT pathways.^[^ ^9^ ^]^ A growing body of evidence suggests that SET is an essential regulator of oncogenesis and tumor progression, with its dysregulation having been linked to multiple pathological processes.^[^ ^10^ ^]^ For example, SET antagonism can reduce pS62‐c‐Myc levels and c‐Myc transcriptional activity and thereby suppress the growth and tumorigenic potential of breast cancer cells.^[^ ^7a^ ^]^ Additionally, SET is associated with poor clinical outcomes and malignant transformation in colorectal cancer,^[^ ^11^ ^]^ non‐small cell lung cancer,^[^ ^12^ ^]^ hepatocellular carcinoma,^[^ ^13^ ^]^ and head and neck squamous cell carcinoma.^[^ ^14^ ^]^ However, the underlying mechanisms whereby SET expression can be negatively regulated remain to be defined.

Proteins in the TRIM (tripartite motif) family have been linked to diverse biological processes owing to their ability to leverage the ubiquitin‐proteasome to induce the degradation of specific target substrate proteins. As such, TRIM dysregulation is a hallmark of many cancers, viral infections, developmental disorders, and neurodegenerative conditions.^[^ ^15^ ^]^ The TRIM family member TRIM4 has recently been shown to be involved in the virus‐induced IFN production,^[^ ^16^ ^]^ oxidative stress‐induced cell death,^[^ ^17^ ^]^ and hepatocellular carcinoma.^[^ ^18^ ^]^ The functional importance of TRIM4 in breast cancer, however, has yet to be established.

Herein, we provide evidence that TRIM4 is downregulated in TAM‐resistant breast cancer cells, and that the loss of TRIM4 expression is associated with hormone receptor‐independent phenotypes, including TAM resistance and triple‐negative breast cancer (TNBC). Mechanistically, TRIM4 enhances ER‐*α* action via mediating the K48‐linked ubiquitination and proteasomal degradation of SET. Moreover, SET impairs p53 and PP2A‐induced ER‐*α* expression, while this can be rescued by TRIM4‐mediated SET degradation. Together, these results reveal a novel mechanism controlling SET degradation and ER‐*α* action in breast cancer cells and suggest that TRIM4 is a promising target for efforts to increase breast cancer cell sensitivity to endocrine therapy.

## 2 Results

### 2.1 TRIM4 is Downregulated in TAM‐Resistant Breast Cancer Cells and Associated with Patient Prognosis

**Figure 1:**
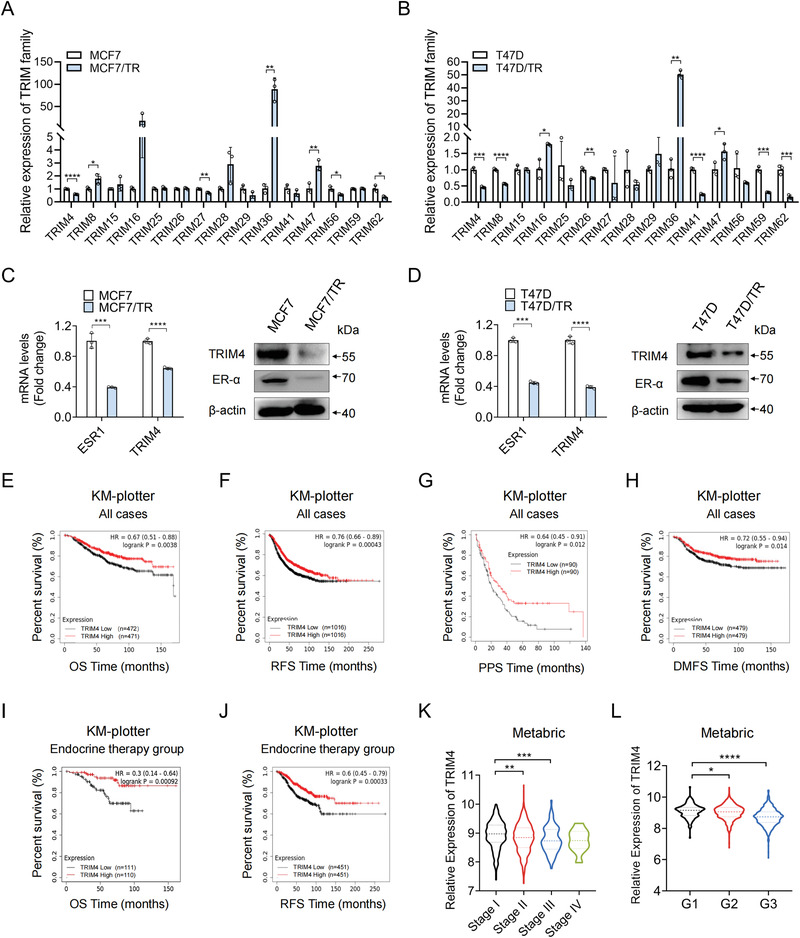
TRIM4 is downregulated in TAM‐resistant breast cancer cells and is associated with patient prognosis. A) TRIM family gene expression in MCF7 and MCF7/TR cells, as assessed via qPCR. B) TRIM family gene expression in T47D and T47D/TR cells, as assessed via qPCR. C) TRIM4 and ER‐*α* levels in MCF7 and MCF7/TR cells were assessed via qPCR and western blotting. D) TRIM4 and ER‐*α* levels in T47D and T47D/TR cells were assessed via qPCR and western blotting. E–H) Overall breast cancer patients’ E) OS, F) RFS, G) PPS, and H) DMFS as a function of TRIM4 expression levels were assessed with the KM‐plotter database. I,J) ER‐*α*‐positive breast cancer I) OS , and J) RFS after endocrine therapy treatment as a function of TRIM4 expression level. K) TRIM4 gene expression levels at different tumor stages of breast cancer cohorts in the metabric dataset (Stage 1: *n* = 371, Stage 2: *n* = 571, Stage 3: *n* = 90, Stage 4: *n* = 10,). L) TRIM4 gene expression levels at different grades of breast cancer cohorts in the metabric dataset (G1: *n* = 170, G2: *n* = 770, G3: *n* = 952). For A–D), representative of three independent experiments. Data information: data were presented as mean ± SD. Unpaired two‐tailed Student's *t*‐test; *p < 0.05; **p < 0.01; ***p < 0.001; ****p < 0.0001.

To evaluate the potential regulatory activity of different TRIM family proteins in the context of breast cancer cell TAM resistance, we queried RNA‐seq data compiled in the GEO database and identified multiple TRIM genes that were differentially expressed in TAM‐resistant T47D (T47D/TR) cells relative to parental T47D cells (Figure S1A, Supporting Information). To confirm these results, we established MCF7/TR and T47D/TR TAM‐resistant cell lines with reduced ER‐*α* expression levels, consistent with many prior studies.^[^ ^3^, ^19^ ^]^ Moreover, we confirmed that TRIM4 expression was downregulated in both of these cell lines relative to its corresponding parental controls (**Figure** **1**A–D), suggesting that it may play a role in the development of TAM resistance.

By analyzing several publicly available gene expression datasets, we found that the overall, relapse‐free, post‐progression, distant metastasis‐free, and disease‐specific survival (OS, RFS, PPS, DMFS, and DS) outcomes for breast cancer patients exhibiting lower levels of TRIM4 expression were significantly worse than those for patients expressing higher levels of this gene in the overall breast cancer patient population (Figure 1E–H and Figure S1B,C, Supporting Information). Moreover, reduced TRIM4 expression was linked to worse OS and RFS outcomes among ER‐*α*‐positive breast cancer patients following endocrine therapy (Figure 1I,J). Low TRIM4 expression was also correlated with poor prognosis in patients with luminal A‐type disease (Figure S1D–F, Supporting Information). Additionally, reduced TRIM4 levels were significantly associated with more advanced stage, higher histological grade, negative ER‐*α* status, and basal‐like subtype (Figure 1K,L and Figure S1G,H, Supporting Information). Together, these findings thus suggested that TRIM4 may be a prognostic biomarker in breast cancer patients, particularly in ER‐*α*‐positive patients undergoing TAM treatment or other endocrine therapies.

### 2.2 Loss of TRIM4 is Associated with TAM Resistance In Vitro and In Vivo

**Figure 2:**
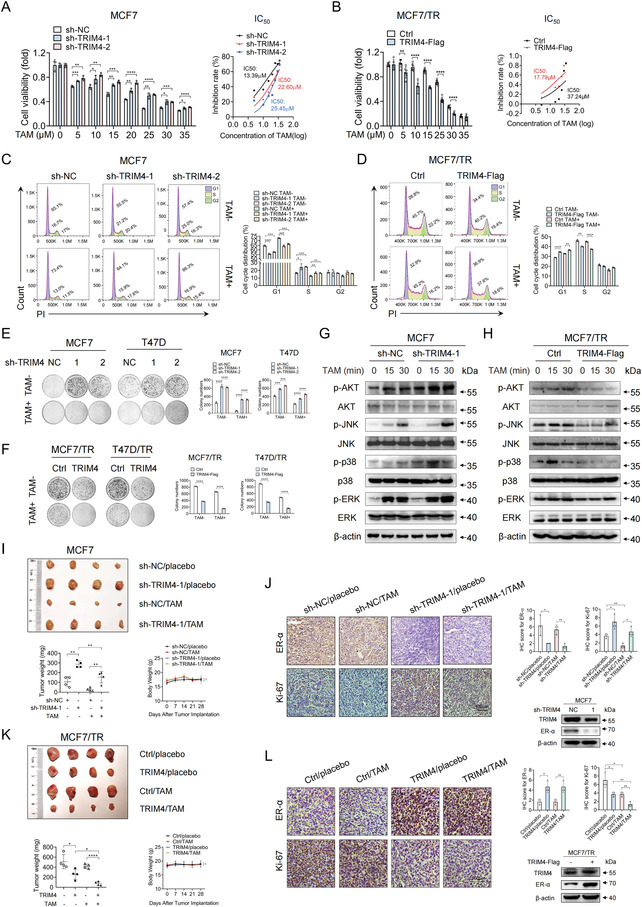
Loss of TRIM4 is associated with TAM resistance in vitro and in vivo. A,B) An MTT assay was used to calculate viability levels and TAM IC_50_ values for A) TRIM4‐silenced or control MCF7 cells and B) TRIM4‐overexpressing or control MCF7/TR cells after treatment for 48 h with the indicated TAM doses (in µM). C,D) Cell cycle progression was assessed via flow cytometry in C) TRIM4‐silenced or control MCF7 cells and in D) TRIM4‐overexpressing or control MCF7/TR cells after treatment with TAM for 48 h. E,F) The colony formation activity of E) TRIM4‐silenced MCF7, T47D and F) TRIM4‐overexpressing MCF7/TR, T47D/TR cells was evaluated following treatment with TAM. G,H) TAM resistance‐related changes in the MAPK and AKT signaling pathways were assessed in G) TRIM4‐silenced or control MCF7 cells and in H) TRIM4‐overexpressing or control MCF7/TR cells via western blotting. I–L) Nude BALB/c mice were subcutaneously implanted with the indicated tumor cells (*n* = 4/group) with some mice having first been implanted with E2 pellets. Mice were then treated for 4 weeks with PBS or TAM (5 mg kg^−1^). I,K) Tumor images, weights and mice body weights were compared among groups. Body weight changes between different groups were assessed by two‐way ANOVA analysis using GraphPad Prism 8.4.2 (GraphPad Prism, RRID: SCR_0 02798); Δ, no significance. J,L) IHC staining and western blotting were conducted to assess intratumoral ER‐*α* (mainly located in the nucleus) and Ki‐67 (mainly located in the nucleus) expression. Representative images of four xenografts per group are shown. Scale bars: 100 µm. IHC scores for ER‐*α* and Ki‐67 were quantified. Data were representative of at least three independent experiments. Data information: data were presented as mean ± SD. Unless otherwise noted, data were analyzed using unpaired two‐tailed Student's *t*‐test; *p < 0.05; **p < 0.01; ***p < 0.001; ****p < 0.0001.

**Figure 3:**
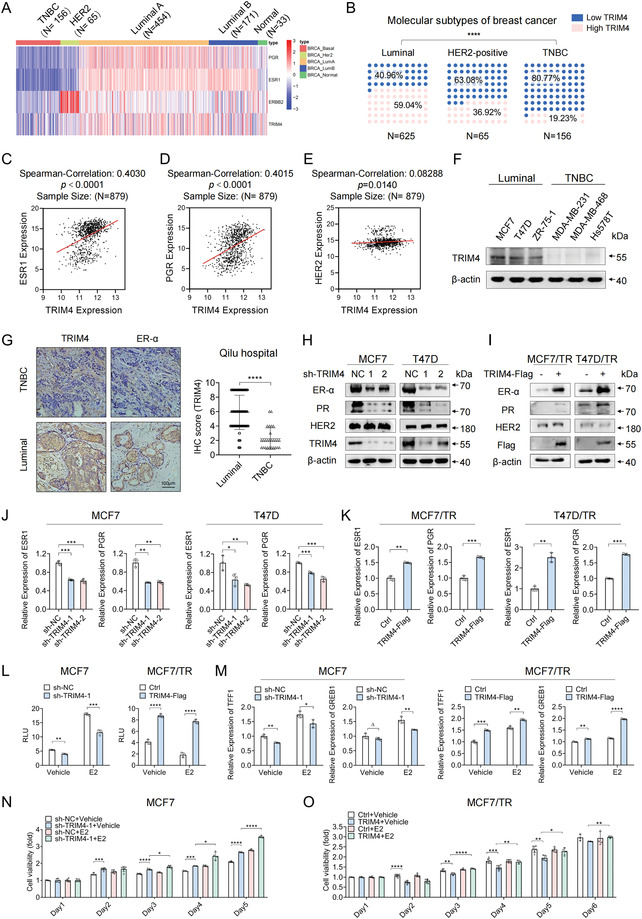
TRIM4 induces ER‐*α* upregulation and promotes ER‐*α*‐dependent transcriptional activity. A) A TCGA data‐based heatmap displaying *ESR1*, *PGR*, *ERBB2*, and *TRIM4* expression levels in TNBC (*n* = 156), HER2 (*n* = 65), luminal A (*n*= 454), luminal B (*n* = 171), and normal breast cancer subtypes (*n* = 33). B) TRIM4 positivity and negativity frequencies in different subtypes of breast cancer were assessed using pie charts. ****p < 0.0001, Fisher's exact test. C–E) Scatter plots were used to demonstrate correlations between the expression of TRIM4 and that of *ESR1*, *PGR*, and *ERBB2* in the TCGA database. Spearman rank correlation analyses were used to establish *r*‐values in each group. F) TRIM4 levels in luminal (MCF7, T47D, and ZR‐75‐1) and TNBC (MDA‐MB‐231, MDA‐MB‐468, and Hs578T) cell lines were assessed via western blotting. G) Representative IHC images and charts demonstrating correlations between the expression of TRIM4 (mainly located in the cytoplasm) and that of ER‐*α* in 143 breast tumor samples (116 ER‐*α*‐positive and 27 TNBC samples). Scale bars: 100 µm. H) The expression of the indicated luminal markers was assessed in TRIM4‐silenced or control luminal cells via western blotting. I) The expression of the indicated luminal markers was assessed in TRIM4‐overexpressing or control TAM‐resistant cells via western blotting. J,K) The expression of *ESR1* and *PGR* was assessed via qPCR in TRIM4‐silenced or control luminal cells and TRIM4‐overpressing TAM‐resistant cells. L) ERE‐luciferase activity was assessed for TRIM4‐silenced or control MCF7 and TRIM4‐overexpression or control MCF7/TR cells with or without E2. M) *GREB1* and *TFF1* expression levels were assessed via qPCR in TRIM4‐silenced or control MCF7 and TRIM4‐overexpression or control MCF7/TR (Q) cells with or without E2. N,O) MTT assays were used to assess the viability of TRIM4‐silenced or control MCF7 and TRIM4‐overexpression or control MCF/TR cells treated with the indicated doses of E2 or ethanol (vehicle) for the indicated amount of time. For all experiments, representative of at least three independent experiments. Data information: data were presented as mean ± SD. Unpaired two‐tailed Student's *t*‐test; *p < 0.05; **p < 0.01; ***p < 0.001; ****p < 0.0001; Δ, no significance.

To determine the functional significance of TRIM4 as a regulator of TAM resistance, we utilized TRIM4‐specific shRNAs and an overexpression plasmid to efficiently knock down and overexpress TRIM4. Sh‐TRIM4‐1 exhibited higher knockdown efficiency of the two tested shRNAs (Figure 3H and Figure S2A–H, Supporting Information). TRIM4 knockdown not only improved TAM IC_50_ values and colony formation activity in MCF7 and T47D cells (**Figure** **2**A,E and Figure S3A, Supporting Information), but also resulted in an increase in the frequency of cells in the S phase of the cell cycle (Figure 2C, and Figure S3E, Supporting Information). In contrast, TRIM4 overexpression in MCF7 and T47D cells increased TAM sensitivity (Figure S3C,D, Supporting Information). Consistently, TRIM4 overexpression in MCF7/TR and T47D/TR cells also restored TAM sensitivity (Figure 2B,F and Figure S3B, Supporting Information) and led to cell cycle arrest at the G1 phase (Figure 2D, and Figure S3F, Supporting Information).

We then investigated the role of TRIM4 in TAM‐mediated AKT and MAPK activation. As shown in Figure S3G, Supporting Information, TAM‐resistant cells exhibited an activation phenotype for both the AKT and MAPK pathways. Loss of TRIM4 expression in MCF7 and T47D cells enhanced the TAM‐mediated phosphorylation of AKT and MAPK family proteins (Figure 2G and Figure S3H, Supporting Information). Conversely, TRIM4 re‐expression in TAM‐resistant cells inhibited their activation (Figure 2H and Figure S3I, Supporting Information).

Furthermore, as shown in Figure S4A,B, Supporting Information, TAM‐resistant cells exhibited increased proliferative activity relative to their parental cells. Loss of TRIM4 increased the proliferation, migration, and invasion of these parental cells (Figure S4C,D,I,J, Supporting Information), while TRIM4 upregulation suppressed all of the above activities in both parental and TAM‐resistant cells (Figure S4E–H,K–N, Supporting Information). Our data also underscored the critical role of TRIM4 in breast cancer stem cells based on the observed proportions of CD44^+^/CD24^−^ cells (Figure S4O,P, Supporting Information). Together, these data suggested that TRIM4 loss was closely tied to breast cancer cell proliferation, metastasis, and TAM resistance.

Next, we explored the functional role of TRIM4 as a regulator of ER‐*α*‐positive breast cancer TAM resistance in a murine xenograft model system. Briefly, nude mice were subcutaneously implanted with MCF7 cells stably expressing TRIM4 shRNA‐1 or sh‐NC, or with MCF7/TR cells stably overexpressing TRIM4 or control constructs. When tumors were palpable, mice were administered TAM or vehicle control. In line with our in vitro results, TRIM4 loss in MCF7 cells was associated with increases in tumor volume relative to controls, while TAM treatment further exaggerated this difference and resulted in a decrease in tumor volume (Figure 2I). Overexpressing TRIM4 in MCF7/TR cells was associated with the restoration of TAM sensitivity (Figure 2K). No significant body weight loss occurred during treatment (Figure 2I,K). IHC staining and western blotting revealed that TRIM4 knockdown suppressed the expression of ER‐*α* while enhancing Ki‐67 expression within xenograft tumors (Figure 2J), whereas TRIM4 upregulation yielded the opposite phenotypes (Figure 2L). To further establish the clinical relevance of TRIM4 as a modulator of TAM sensitivity in breast cancer patients, we next prepared organoids derived from ER‐*α*‐positive patients which were then infected using TRIM4‐expressing or control retroviruses and plated in organoid culture media and Matrigel. The overexpression of TRIM4 in these organoids not only reduced the number and size thereof (Figure S5A, Supporting Information) but also increased their TAM sensitivity (Figure S5B, Supporting Information). The stability of the organoids is shown in Figure S5C, Supporting Information. Together, these results confirmed the ability of TRIM4 to suppress breast cancer development and resistance to TAM treatment.

### 2.3 TRIM4 Induces ER‐*α* Upregulation and Promotes ER‐*α*‐Dependent Transcriptional Activity

A loss of hormone receptor expression is one of the primary drivers of resistance to antihormonal therapy in breast cancer patients.^[^ ^3d^ ^]^ An analysis of available TCGA data indicated that TRIM4 expression was closely related to luminal subtypes of breast cancer but not to TNBC (**Figure** **3**A,B). Specifically, there was a positive correlation between the expression of TRIM4 and that of *PGR*, *ESR1*, *GATA3*, and *FOXA1* (*p* < 0.0001, respectively), which are markers of luminal disease, while it was not correlated with the expression of *HER2* (*p* = 0.0140) (Figure 3C–E and Figure S6A–C, Supporting Information). Consistently higher levels of TRIM4 protein expression were evident in luminal breast cancer cell lines as compared to TNBC cell lines (Figure 3F). We further conducted an IHC analysis of the protein levels of ER‐*α* and TRIM4 in primary human breast tumor samples collected at Qilu hospital, revealing markedly lower levels of intratumoral expression in patients with TNBC as compared to patients with luminal‐type breast cancer (Figure 3G). These data indicated that the expression of TRIM4 was associated with hormone receptor positivity, while the loss of such expression was closely related to TNBC incidence.

Next, we evaluated the impact of TRIM4 on these hormone receptors. In line with gene expression array results, TRIM4 knockdown resulted in decreases in ER‐*α* and PR expression in the MCF7 and T47D luminal breast cancer cell lines (Figure 3H,J). The overexpression of TRIM4 increased ER‐*α* expression levels in the MCF7/TR and T47D/TR cells as well as in MDA‐MB‐231 and MDA‐MB‐468 TNBC cell lines (Figure 3I and Figure S6F, Supporting Information), while changes in HER2 expression were not observed following the modulation of TRIM4 expression in any of these tested cell lines (Figure 3H,I and Figure S6F, Supporting Information). TRIM4 overexpression also increased ER‐*α* and PR mRNA levels in MCF7/TR and T47D/TR cells (Figure 3K). Together, these results suggested that TRIM4 could modulate ER‐*α* expression at the mRNA and protein levels.

We further sought to determine whether the TRIM4‐mediated regulation of ER‐*α* expression also altered ER‐*α*‐dependent transcription in breast cancer cells. Following 17*β*‐estradiol (E2) stimulation, TRIM4 expression levels were slightly increased in both T47D and MCF7 cells (Figure S6G, Supporting Information). Knocking down TRIM4 attenuated E2‐induced estrogen‐response element (ERE) luciferase reporter activity and the expression of the ER‐*α* target genes TFF1 and GREB1 in MCF7 and T47D cells (Figure 3L,M and Figure S6H,I, Supporting Information). Conversely, the overexpression of TRIM4 increased such E2‐induced ERE activity and gene expression in MCF7/TR and MDA‐MB‐231 cells (Figure 3L,M and Figure S6J, Supporting Information). Loss of TRIM4 expression increased MCF7 cell growth irrespective of E2 treatment (Figure 3N), while TRIM4 overexpression slowed MCF7/TR and MDA‐MB‐231 cell growth, and E2 treatment alleviated such growth retardation (Figure 3O and Figure S6K, Supporting Information), suggesting that TRIM4 overexpression may restore E2 signaling to TAM‐ resistant and TNBC cells.

### 2.4 TRIM4 Targets SET

**Figure 4:**
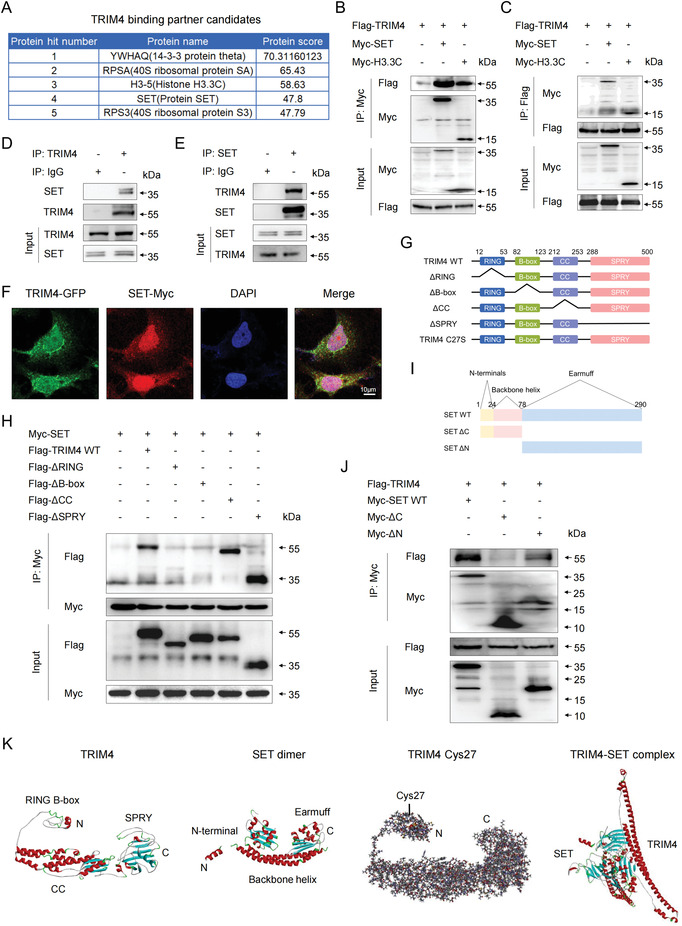
TRIM4 targets SET. A) Candidate TRIM4 binding proteins identified via a mass spectrometry‐based cross‐linking assay. B,C) HEK293T cells were transfected with Flag‐TRIM4 and the indicated Myc‐tagged plasmids, after which immunoprecipitation (IP) was performed to assess interactions between TRIM4, SET, and H3.3C using B) anti‐Myc or C) anti‐Flag antibodies; IB, immunoblot. D,E) The ability of endogenous SET to co‐immunoprecipitate with endogenous TRIM4 in MCF7 cell lysates was assessed using D) anti‐TRIM4 or E) anti‐SET for IP. F) An immunofluorescent approach was used to evaluate the colocalization of TRIM4 and SET within MCF7 cells. Scale bar: 20 µm. G) Schematic overview of TRIM4 and prepared TRIM4 truncation mutants. RING, RING domain; B‐box, B box domain; CC, coiled‐coil domain; SPRY, PRY/SPRY domain. H) HEK293T cells were transfected with Flag‐tagged TRIM4 mutants and Myc‐SET, after which anti‐Myc was used to immunoprecipitate these lysates, followed by immunoblotting with the indicated antibodies. I) Schematic of SET and its truncation mutants. J) HEK293T cells were transfected with Myc‐tagged SET mutants and TRIM4‐Flag, after which anti‐Myc was used to immunoprecipitate these lysates, followed by immunoblotting with the indicated antibodies. K) I‐TASSER (I‐TASSER, RRID: SCR_014627) was used to generate 3D structures for TRIM4 and SET, the TRIM4‐SET complex was predicted using ZDOCK (v 3.0.2), and TRIM4 Cys27 models were constructed using Discovery Studio (v 4.5). All data were representative of n = 3 independent experiments.

**Figure 5:**
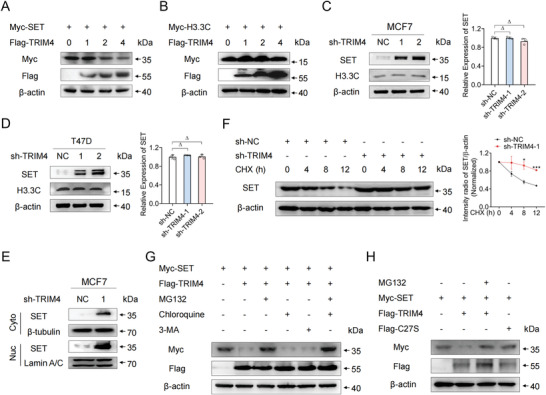
TRIM4 induces the proteasomal degradation of SET. A,B) Immunoblotting was performed to analyze lysates prepared from HEK293T cells following transfection with A) Myc‐SET or B) Myc‐H3.3C and different doses of the Flag‐TRIM4 expression plasmid (0, 1, 2, or 4 µg). C,D) Immunoblotting and qPCR were performed to evaluate the expression of SET and H3.3C in TRIM4‐silenced or control C) MCF7 and D) T47D cells. E) SET expression in the nuclear and cytoplasmic fractions of TRIM4‐silenced or control MCF7 cells was assessed via western blotting. F) MCF7 cells were transfected using the indicated plasmid, followed by cycloheximide (CHX) treatment for the indicated duration. Immunoblotting was then performed using lysates prepared from these cells, with the ImageJ software being used to quantify SET expression and *β*‐actin being used for normalization. G) Following transfection with the Flag‐TRIM4 and Myc‐SET plasmids and treatment with 3‐MA, chloroquine, or MG132 for 4 h, HEK293T lysates were subjected to western blotting. H) After transfection with expression plasmids for Myc‐SET and either Flag‐TRIM4 or Flag‐TRIM4 C27S, HEK293T cell lysates were subjected to western blotting. All data were representative of *n* = 3 independent experiments. Data information: data were presented as mean ± SD. Unpaired two‐tailed Student's *t*‐test; *p < 0.05; ***p < 0.001; Δ, no significance.

To clarify the targets of TRIM4 within cells, we next used MCF7 cells to conduct a mass spectrometry‐based formaldehyde cross‐linking assay which identified SET and H3.3C as putative TRIM4 binding partners (**Figure** **4**A and Figure S7A, Supporting Information). TRIM4 was further confirmed to interact with SET or H3.3C in HEK293T cells (Figure 4B,C). TRIM family members have been shown to exhibit E3 ubiquitin ligase activity that enables them to induce the proteasomal degradation of specific substrates.^[^ ^15b^ ^]^ We therefore next explored the ability of TRIM4 to regulate SET and H3.3C expression. While TRIM4 was able to suppress SET expression, it had no impact on H3.3C expression (**Figure** **5**A,B), implying that TRIM4 may target SET. Endogenous interactions between SET and TRIM4 were additionally detected within MCF7 cells (Figure 4D,E), and these proteins colocalized in MCF7 cells (Figure 4F). An analysis of the KM‐plotter database revealed SET expression to be associated with worse ER‐*α*‐positive breast cancer patient OS, RFS, and DMFS following TAM treatment (Figure S8A–C, Supporting Information). Moreover, SET upregulation in MCF7/TR and T47D/TR cells resulted in increased TAM IC_50_ values (Figure S8D,E, Supporting Information). Together, these results support the identification of SET as a TRIM4 target protein.

TRIM4 contains a RING finger, B‐box, coiled‐coil, and B30.2/SPRY domains (Figure 4G,K). To determine which of these domains enable TRIM4 to interact with SET, a series of TRIM4 truncation mutants were generated (Figure 4G). SET was able to co‐precipitate with wild‐type (WT) TRIM4, coiled‐coil domain deletion mutant (ΔCC) TRIM4, and SPRY domain deletion mutant (ΔSPRY) TRIM4, but not RING domain deletion mutant (ΔRING) or B‐box domain deletion mutant (ΔB‐box) TRIM4 (Figure 4H). These data suggest that the N‐terminal domain of TRIM4 interacts with SET. SET primarily exists in a dimerized form in a shape reminiscent of headphones (Figure 4K), with each subunit consisting of an N‐terminus, a backbone helix, and an earmuff domain ^[^ ^20^ ^]^ (Figure 4I,K). When co‐immunoprecipitation experiments were performed with SET truncation mutants, the SET earmuff domain was necessary for TRIM4 interactions (Figure 4J). Three‐dimensional TRIM4, SET, and TRIM4‐SET complexes are shown in Figure 4K.

### 2.5 TRIM4 Induces the Proteasomal Degradation of SET

The overexpression of TRIM4 in HEK293T cells decreased SET protein levels therein in a dose‐dependent fashion, whereas the same was not true for H3.3C (Figure 5A,B). Consistent with these results, reductions in TRIM4 expression in MCF7 and T47D cells adversely impacted SET protein levels without impacting SET mRNA levels in these cells (Figure 5C,D). Obviously, our results indicated that TRIM4 was mainly involved in the process of post‐translational modification, but had no effect on mRNA transcription of SET (Figure 5C,D). Nuclear and cytoplasmic separation assays were additionally conducted, revealing that SET expression in both the nuclear and cytoplasmic fractions was increased by TRIM4 knockdown (Figure 5E). Moreover, CHX chase assays demonstrated that the knockdown of TRIM4 in MCF7 cells extended the half‐life of the SET protein (Figure 5F), while TRIM4 overexpression promoted SET protein degradation in HEK293T cells (Figure S9A, Supporting Information). The proteasome inhibitor MG132 was sufficient to reverse the observed TRIM4‐induced SET degradation, whereas chloroquine and 3‐methyladenine (3‐MA), which respectively inhibit lysosome activity and the autophagy pathway, had no impact on such degradation (Figure 5G). A TRIM4 point mutation (C27S) involving the substitution of the cysteine residue at position 27 with a serine ^[^ ^16^ ^]^ disrupted the ability of this protein to induce SET degradation (Figure 5H). Together, these results provided strong evidence that the E3 ubiquitin ligase activity of TRIM4 could induce the proteasomal degradation of SET.

### 2.6 TRIM4 Promotes SET K48‐Polyubiquitination

**Figure 6:**
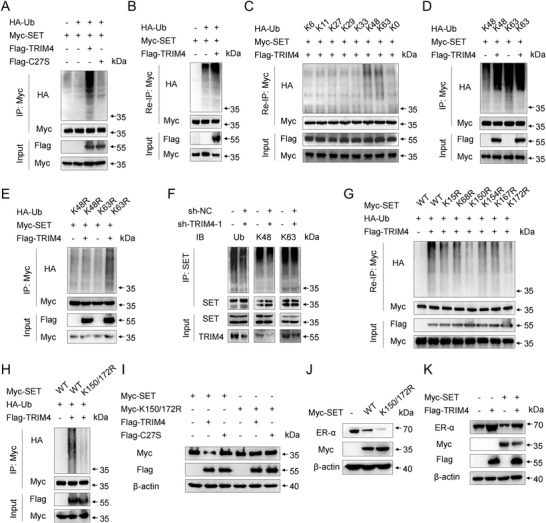
TRIM4 promotes SET K48‐polyubiquitination. A) Anti‐Myc was used to immunoprecipitate lysates prepared from HEK293T cells following transient HA‐Ub, Myc‐SET, and TRIM4‐Flag or TRIM4 C27S‐Flag co‐transfection, after which anti‐HA was used for immunoblotting. B) Lysates from HEK293T cells transiently cotransfected with HA‐Ub, Myc‐SET, and Flag‐TRIM4 were subjected to immunoprecipitation with an anti‐Myc antibody. The immunoprecipitates were denatured and re‐immunoprecipitated with anti‐Myc (two‐step immunoprecipitation, Re‐IP) and then analyzed via western blotting. C) Re‐IP lysates from HEK293T cells transiently cotransfected with HA‐Ub (WT and its mutants), Flag‐TRIM4, and Myc‐SET. D) Immunoprecipitation analyses were performed for lysates from HEK293T cells following the transient co‐transfection of Myc‐SET, Flag‐TRIM4, and K48‐Ub or K63‐Ub mutant. E) Immunoprecipitation analyses were performed for lysates from HEK293T cells following Myc‐SET, Flag‐TRIM4, and K48R‐Ub or K63R‐Ub mutant transient co‐transfection. F) Anti‐SET was used to immunoprecipitate TRIM4‐silenced or control MCF7 cell lysates, after which immunoblotting was performed using the indicated antibodies. G,H) Immunoprecipitation analyses were performed for lysates prepared from HEK293T cells following HA‐Ub, Flag‐TRIM4, and Myc‐SET (WT or point mutants) co‐transfection. I) Immunoblotting analyses were performed for lysates prepared from HEK293T cells following co‐transfection with Flag‐TRIM4 or the TRIM4 C27S expression plasmid and Myc‐SET or the Myc‐SET K150/172R mutant. J) MCF7 cells were transfected with SET‐Myc or Myc‐SET K150/172R mutant constructs for 48 h, after which immunoblotting of lysates prepared from these cells was conducted using the indicated antibodies. K) MCF7 cells were cotransfected with Myc‐SET and TRIM4, after which immunoblotting of lysates prepared from these cells was conducted using the indicated antibodies. All data were representative of *n* = 3 independent experiments.

Ubiquitination is a post‐translational modification that enables proteins to be degraded by the ubiquitin‐proteasome degradation pathway.^[^ ^21^ ^]^ We, therefore, evaluated the ability of TRIM4 to induce SET ubiquitination. When HEK293T cells were transfected with a WT plasmid, SET polyubiquitination was increased, whereas no such change was evident following TRIM4 C27S mutant plasmid transfection (**Figure** **6**A). Given that TRIM4 or SET may form a complex with other nonspecific proteins, we performed a two‐step immunoprecipitation assay (Re‐IP) to reduce the presence of nonspecific associated proteins, revealing similar results (Figure 6B). To better classify the TRIM4‐induced SET polyubiquitin chain linkage on SET, a series of ubiquitin mutant isoforms were established in which all lysine (K) residues other than the indicated lysine were sequentially substituted for arginine (R). SET polyubiquitination was evident for cells transfected with the K48 or K63 plasmid, whereas it was not evident for other mutants (Figure 6C). Moreover, TRIM4 markedly enhanced the K48‐linked polyubiquitination of SET but had no impact on SET K63‐linked polyubiquitination (Figure 6D). When these ubiquitin assays were repeated using ubiquitin mutant constructs harboring a lysine‐to‐arginine substitution at either the K48 or K63 position (K48R or K63R), TRIM4‐mediated SET ubiquitination was evident for the K63R but not the K48R isoform of this protein (Figure 6E). To further confirm the ability of TRIM4 to induce endogenous SET polyubiquitination, SET ubiquitination was assessed in MCF7 cells, revealing that the deletion of TRIM4 selectively inhibited SET K48‐linked polyubiquitination but not SET K63‐linked polyubiquitination (Figure 6F). Together, these results provided clear evidence that TRIM4 could promote selective SET K48‐linked polyubiquitination.

Next, we sought to identify ubiquitinated lysine (K) residues in SET. The Protein Lysine Modification Database predicted the presence of six potential K ubiquitination sites in SET (Figure S7B,C, Supporting Information). We then replaced each of these lysine residues with arginine (R) to respectively generate the K15R, K68R, K150R, K154R, K167R, and K172R SET mutant proteins. Subsequent Re‐IP assays revealed that the K150R and K172R mutations partially disrupted TRIM4‐mediated SET ubiquitination (Figure 6G). We then generated a K150/K172R double mutant SET construct and found that TRIM4‐mediated ubiquitination and associated SET degradation were wholly absent for this mutant protein (Figure 6H,I). Relative to WT SET, the K150/K172R mutation significantly suppressed the expression of ER‐*α* in MCF7 cells (Figure 6J). As shown in Figure 6K, TRIM4 rescued the inhibition of ER‐*α* expression induced by SET overexpression in MCF7 cells. Together, these results thus suggested that K150 and K172 are essential residues for SET ubiquitination and degradation.

### 2.7 TRIM4 Reverses TAM Resistance in Breast Cancer Partially Mediated by SET

**Figure 7:**
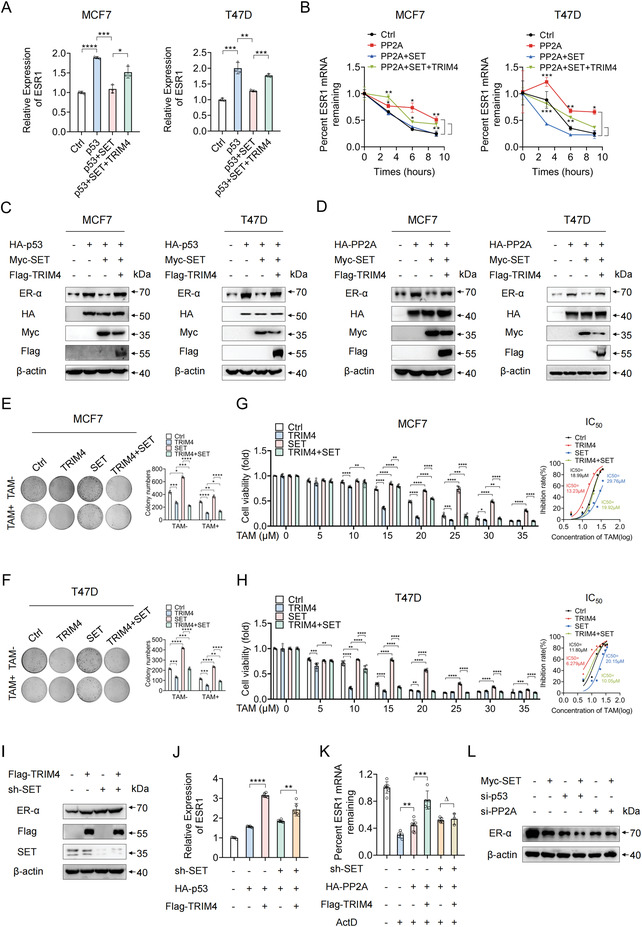
TRIM4 reverses TAM resistance in breast cancer partially mediated by SET. A) MCF7 and T47D cells were co‐transfected with HA‐tagged p53, Myc‐SET, and Flag‐TRIM4, after which the expression levels of *ESR1* were assessed via qPCR. B) MCF7 and T47D cells were co‐transfected with HA‐tagged PP2A, Myc‐SET, and Flag‐TRIM4, and after the indicated duration of ActD treatment, the expression levels of *ESR1* were assessed via qPCR. C) MCF7 and T47D cells were co‐transfected with HA‐tagged p53, Myc‐SET, and Flag‐TRIM4, after which immunoblotting of lysates prepared from these cells was conducted using the indicated antibodies. D) MCF7 and T47D cells were co‐transfected with HA‐tagged PP2A, Myc‐SET, and Flag‐TRIM4, after which immunoblotting of lysates prepared from these cells was conducted using the indicated antibodies. E,F) Representative images of colonies formed by E) MCF7 and F) T47D cells expressing indicated plasmids after treatment with TAM (5 µM) for 24 h. G,H) MTT assays were used to calculate viability levels and TAM IC_50_ values for G) MCF7 and H) T47D cells transfected as indicated. I) Sh‐NC or sh‐SET was co‐transfected with pCDNA3.1‐Flag or TRIM4‐Flag vectors into MCF7 cells, after which the immunoblotting of lysates prepared from these cells was conducted using the indicated antibodies. J) Sh‐NC or sh‐SET were co‐transfected with HA‐tagged p53 and Flag‐tagged TRIM4, after which the expression levels of *ESR1* were assessed via qPCR. K) Sh‐NC or sh‐SET were co‐transfected with HA‐tagged PP2A and Flag‐tagged TRIM4, and the expression levels of *ESR1* were assessed via qPCR following a 6 h ActD treatment. L) MCF7 cells were co‐transfected with si‐p53 or si‐PP2A and SET‐myc, after which the immunoblotting of lysates prepared from these cells was conducted using the indicated antibodies. All data were representative of *n* = 3 independent experiments. Data information: data were presented as mean ± SD. Unpaired two‐tailed Student's *t*‐test; *p < 0.05; **p < 0.01; ***p < 0.001; ****p < 0.0001; Δ, no significance.

We next assessed whether SET is involved in TRIM4‐mediated TAM sensitivity in breast cancer. As shown in **Figure** **7**A,C, SET suppressed p53‐induced ER‐*α* expression while this was reversed by TRIM4. Given that PP2A has been reported to mediate *ESR1* mRNA stability,^[^ ^5^ ^]^ we performed an Actinomycin D assay which revealed that SET impaired the PP2A‐mediated stabilization of the *ESR1* mRNA, while this was also reversed by TRIM4 (Figure 7B). Consistently, SET impaired PP2A‐mediated increases in ER‐*α* protein levels, while TRIM4 reversed this phenotype (Figure 7D). Moreover, functional assays revealed that TRIM4 overexpression not only impaired SET‐induced colony formation but also altered the corresponding IC_50_ values in MCF7 and T47D cells (Figure 7E–H). To further demonstrate the potential roles of SET in TRIM4 mediated ER‐*α* expression and TAM sensitivity, we used an shRNA system to knock down SET expression in MCF7 cells, demonstrating that the enhancement of ER‐*α* protein expression and TAM sensitivity mediated by TRIM4 were attenuated under conditions of SET knockdown (Figure 7I and Figure S8F,G, Supporting Information). Furthermore, a CHX chase assay revealed that TRIM4 and SET expression levels had no significant impact on ER‐*α* degradation (Figure S9B, Supporting Information). To further test whether TRIM4 directly regulates the functions of p53 and PP2A, a series of transfection and immunoprecipitation experiments were performed. These analyses revealed that TRIM4 was potentially able to interact with p53 and PP2A (Figure S9E, Supporting Information), but these interactions appeared to be unstable as they were no longer detectable when immunoprecipitation and immunoblotting antibodies were exchanged (Figure S9F, Supporting Information), suggesting that TRIM4 may interact with p53 and PP2A through SET. Additionally, TRIM4 enhanced the functions of p53 and PP2A on *ESR1* mRNA expression, and this enhancement was also attenuated by SET knockdown, particularly for PP2A (Figure 7J–K). These results confirmed that the TRIM4‐mediated roles of p53 and PP2A as regulators of ER‐*α* expression were mediated via the SET pathway. As shown in Figure 7L, when p53 was knocked down, SET was still able to inhibit ER‐*α* expression in MCF7 cells. However, when PP2A was knocked down, the inhibition of ER‐*α* expression induced by SET was largely rescued, confirming that PP2A was the main target of SET in this system. As the expression of TRIM4 inhibited the phosphorylation of PI3K family proteins, we wanted to further demonstrate the role of the PI3K signaling pathway in TRIM4‐mediated TAM sensitivity and breast cancer cell proliferation. As shown in Figure S9C,D, Supporting Information, the TRIM4‐PI3K signaling pathway was primarily involved in cell proliferation but not in the regulation of TAM sensitivity.

### 2.8 TRIM4 and SET are Inversely Associated with the Prognosis and Clinicopathological Characteristics of ER‐*α* Positive Breast Cancer Patients

**Figure 8:**
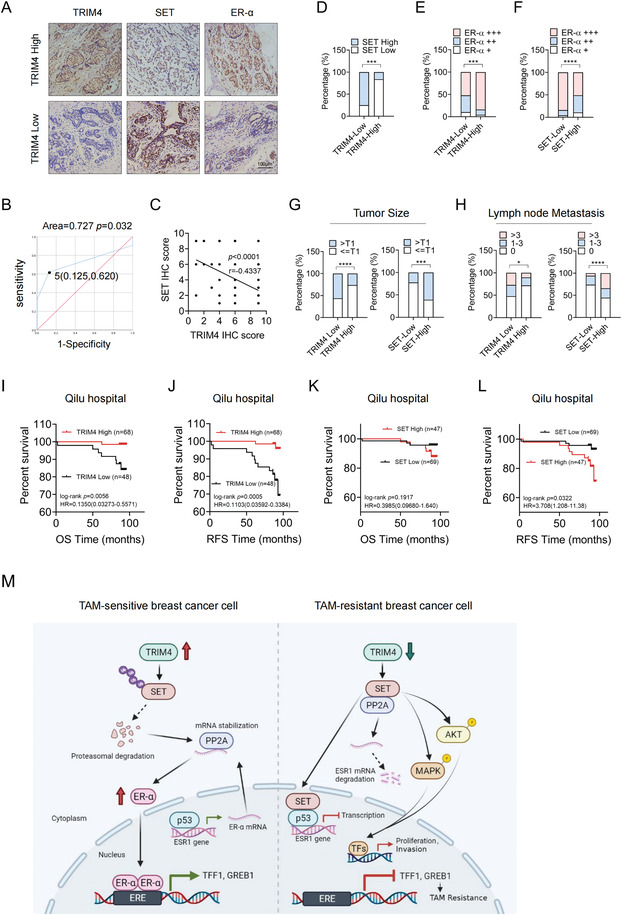
TRIM4 and SET are inversely associated with the prognosis and clinicopathological characteristics of ER‐*α* positive breast cancer patients. A) Representative IHC images and corresponding quantification for analyses of correlations between the expression of TRIM4 and that of SET (widely distributed in the nucleus and the cytoplasm) and ER‐*α* in breast tumor samples from 116 patients with ER‐*α* ‐positive disease. Scale bar, 100 µm. B) The cut‐off used to differentiate between low and high levels of TRIM4 expression was selected with a ROC curve. C) Relationships between TRIM4 and SET expression levels in IHC analyses were assessed via *Spearman's correlation analyses*. D) SET and E) ER‐*α* distributions in TRIM4‐high (*n* = 68) or TRIM4‐low (*n* = 48) groups, as determined based on staining index values. F) ER‐*α* protein expression in SET‐high (*n* = 47) or SET‐low (*n* = 69) groups. G) Tumor size and H) lymph node metastasis distributions as determined in the TRIM4‐high/low or the SET‐high (*n* = 47) or SET‐low (*n* = 69) groups based on staining index values. *n* = 116, *p* values were determined via two‐tailed c*hi‐squared tests*. I,J) ER‐*α*‐positive breast cancer patient I) OS and J) RFS in individuals exhibiting high (*n* = 68) and low (*n* = 48) levels of TRIM4 expression. K,L) ER‐*α* ‐positive breast cancer patient K) OS and L) RFS in patients exhibiting high (*n* = 67) and low (*n* = 69) levels of SET expression. Survival outcomes were analyzed using Kaplan–Meier plots and log‐rank tests. M) Schematic representation of the mechanisms whereby TRIM4 knockdown confers TAM‐resistance in human breast cancer. In breast cancer cells expressing high levels of TRIM4 (left), TRIM4 facilitates SET ubiquitin‐mediated degradation and promotes p53 and PP2A induced ER‐*α* expression, leading to TAM sensitivity. In cells expressing low levels of TRIM4 (right), TRIM4 knockdown enhances SET expression. SET interacts with p53 and PP2A, impairs p53 or PP2A mediated ER‐*α* expression, and activates AKT and MAPK signaling, resulting in estrogen‐independent tumor growth and a TAM‐resistant phenotype. Data information: *p < 0.05; ***p < 0.001; ****p < 0.0001.

To establish the clinical relevance of these findings, TRIM4 and SET protein levels were next assessed in 116 primary tumor tissue samples collected from ER‐*α* positive breast cancer patients undergoing adjuvant TAM treatment at Qilu Hospital. The staining index (SI) score cut‐off value used to differentiate between low and high levels of TRIM4 protein expression in this cohort was calculated using a receiver operating characteristic curve (ROC), with an SI of 5 being optimal to differentiate between TRIM4‐high and TRIM4‐low groups (**Figure** **8**B). TRIM4‐high patients exhibited reduced SET and increased ER‐*α* expression, whereas the opposite was observed for TRIM4‐low patients (Figure 8A,D,E). Spearman correlation analyses revealed TRIM4 and SET expression to be negatively correlated (*r* = ‐0.4337, *p* < 0.0001) (Figure 8C), and the expression of SET and ER‐*α* was similarly negatively correlated (Figure 8F). Together, these data indicate that TRIM4 can regulate SET and ER‐*α* expression in vivo.

Next, the associations between SET or TRIM4 expression and breast cancer patient clinicopathological characteristics were assessed. Lower TRIM4 and higher SET expression levels, respectively, were related to larger tumor size and lymph node metastasis (Figure 8G,H), whereas they were unrelated to age, histological grade, HER‐2, PGR, or Ki‐67 expression (**Table** **1** and Table S2, Supporting Information). Survival analyses revealed increased TRIM4 protein levels to be associated with longer patient OS and RFS (Figure 8I,J), whereas higher levels of SET expression were linked to shorter RFS (Figure 8L), and were unrelated to patient OS (Figure 8K). Univariate and multivariate Cox proportional hazards regression analyses identified only TRIM4 expression as an independent predictor of ER‐*α* positive breast cancer patient OS and RFS (**Table** **2** and Table S3, Supporting Information). Together, these results suggest that decreased TRIM4 expression is linked to TAM resistance and poor prognostic outcomes for ER‐*α* positive breast cancer patients undergoing adjuvant TAM therapy, in line with our results from in vitro studies using TAM‐resistant breast cancer cell lines.

**Table 1:** Correlations between TRIM4 expression and clinical parameters in 116 ER‐*α* positive breast cancer patients

Characteristics	TRIM4‐low (*n* = 48)	TRIM4‐high (*n* = 68)	*p* value
Age			
< 59	38 (79.17%)	54 (79.41%)	> 0.9999
> = 59	10 (20.83%)	14 (20.58%)	
Tumor size			
< = T1	21 (43.75%)	45 (66.18%)	
> T1	27 (56.25%)	16 (23.53%)	0.0017
Unexamined	0	7 (10.29%)	
LN metastasis			
0	23 (47.92%)	49 (72.06%)	
1–3	12 (25.00%)	12 (17.65%)	0.0185
> 3	13 (27.08%)	7 (10.29%)	
Histologic grade			
G1	3 (6.25%)	6 (8.82%)	
G2	29 (60.42%)	42 (61.76%)	0.0983
G3	11 (22.92%)	5 (7.35%)	
Unexamined	5 (10.42%)	15 (22.06%)	
HER‐2			
Neg	33(68.75%)	51(75.00%)	0.5548
Pos	4(8.33%)	4(5.88%)	
Unexamined Ki‐67	11 (22.92%)	13(19.12%)	
< = 14%	12 (25.00%)	26 (38.24%)	0.1075
> 14%	36 (75.00%)	40 (58.82%)	
Unexamined	0	2 (2.94%)	
PR			
Neg	6 (12.50%)	7 (10.29%)	0.7107
Pos	42 (87.50%)	61 (89.71%)	

Data are *n* (%)

All patients were divided into TRIM4 low (SI ≤ 4, *n* = 48) and TRIM4 high (SI > 4, *n* = 68) groups. Clinical variables including, patient age, tumor size, LN (lymph node) metastasis, histological grade, and the expression of HER‐2 and Ki‐67 were used as categorical variables, with corresponding frequencies being calculated accordingly. *p* values were determined via a two‐tailed chi‐square test.

**Table 2:** The effects of different variables on the RFS of 116 breast cancer patients in univariate and multivariate Cox proportional hazards regression model analyses

Variable	Univariate analysis		Multivariate analysis	
	HR (95% CI)	*p*	HR (95% CI)	*p*
Age (> = 59 vs < 59)	2.732 (0.892–8.370)	0.078	–	–
Histologic grade				
G2 versus G1	28 953.213(0.000–3.346E+138)	0.948	–	–
G3 versus G1	64 320.285 (0.000–7.439E+138)	0.944	–	–
Unknown versus G1	12 428.989 (0.000–1.444E+138)	0.952	–	–
Tumor size (>T1 vs < = T1+ Uknown)	1.440 (0.483–4.295)	0.513	–	–
LN metastasis				
1–3 versus 0	2.576 (0.572–11.590)	0.218	1.964 (0.430–8.972)	0.384
>3 versus 0	6.186 (1.743–21.955)	0.005	3.646 (0.962–13.825)	0.057
PR status (pos vs neg)	1.493 (0.194–11.493)	0.700	–	–
HER‐2 status (pos vs neg + unknown)	1.306 (0.168–10.173)	0.799	–	–
Ki‐67 status (pos vs neg + unknown)	1.830 (0.503–6.656)	0.359	–	–
ER‐*α* expression				
++ versus +	35 325.546(0.000–2.949E+149)	0.951	–	–
+++ versus +	22 432.128 (0.000–1.871E+149)	0.953	–	–
SET expression	3.740 (1.148–12.190)	0.029	0.923 (0.238–3.583)	0.908
TRIM4 expression	0.109 (0.024–0.494)	0.004	0.143 (0.026–0.777)	0.024

Variables included patient age, tumor size, LN (lymph node) metastasis, histological grade, and the expression of ER‐*α*, HER‐2, Ki‐67, TRIM4, and SET. Variables were analyzed using a univariate Cox regression model, and the *p* values were determined through univariate Cox regression analyses. For the multivariate Cox regression analysis, histological grade, tumor size, and HER‐2 and Ki‐67 status were excluded (*p* > 0.05), while those variables that were significant in univariate analyses (*p* < 0.05) were incorporated into the multivariate Cox regression model, with *p* values then being calculated via a multivariate Cox regression analysis. No adjustments were made for multiple comparisons.

## 3 Discussion

Resistance to TAM and other endocrine therapies is a major clinical challenge that hinders breast cancer treatment. ER‐*α* expression levels are an important clinical biomarker used to predict TAM treatment outcomes, with a lack of ER‐*α* leading to TAM resistance and TNBC.^[^ ^3^, ^22^ ^]^ Here, we found that TRIM4 serves as a predictor of breast cancer and that the loss of TRIM4 conferred TAM resistance in ER‐*α*‐positive breast cancer. Through analyses of TCGA data, we found that TRIM4 was positively correlated with hormone receptor expression (*ESR1* and *PGR*, but not *ERBB2*), and our results further supported this finding. Moreover, TRIM4 deletion induced estrogen‐independent signaling activity and promoted hormone independence. Specifically, TRIM4 overexpression not only enhanced estrogen receptor signaling in the MCF7/TR and T47D/TR cell lines but also resulted in the re‐expression of ER‐*α* in MDA‐MB‐231 and MDA‐MB‐468 cells. Furthermore, we found that the overexpression of TRIM4 inhibited the proliferation of TNBC cells. It has been reported that the regulation of ER‐*α* transcription is controlled by multiple transcription factors including ERBF‐1,^[^ ^23^ ^]^ AP2,^[^ ^24^ ^]^ FOXO3a,^[^ ^25^ ^]^ FOXM1,^[^ ^26^ ^]^ GATA‐3,^[^ ^27^ ^]^ and p53.^[^ ^4^, ^28^ ^]^ Moreover, several RNA‐binding proteins such as AUFp45,^[^ ^29^ ^]^ HuR,^[^ ^30^ ^]^ and PP2A play critical roles in stabilizing the ER‐*α* mRNA.^[^ ^5^, ^31^ ^]^ We found that SET inhibited p53‐ and PP2A‐mediated ER‐*α* expression, while this could be rescued by TRIM4‐induced SET degradation. Furthermore, knocking down SET expression resulted in TRIM4 dysfunction in breast cancer cells.

ER‐*α* downregulation and TAM treatment also activate several other signaling pathways, including AKT and MAPK signaling, which facilitate cell proliferation and TAM resistance.^[^ ^22^, ^32^ ^]^ MAPK hyperactivation results in the downregulation of ER‐*α* and ER‐*α*‐responsive genes.^[^ ^33^ ^]^ Reciprocally, the inhibition of hyperactive MAPK and AKT signaling pathways results in the restoration of functional ER‐*α* protein expression and endocrine treatment responsiveness.^[^ ^34^ ^]^ Given that PP2A is a negative regulator of MAPK signaling,^[^ ^9c^ ^]^ as an intracellular PP2A inhibitor, SET activates the MAPK pathway.^[^ ^9^, ^35^ ^]^ Our experiments revealed that TRIM4‐mediated SET degradation also plays a critical role in the inhibition of the AKT and MAPK signaling pathways irrespective of TAM treatment status.

Ubiquitin, a small evolutionarily conserved 76 amino acid protein, is found in all eukaryotic tissues,^[^ ^36^ ^]^ wherein it acts as a modifier that covalently attaches to target proteins through an enzymatic cascade in a process known as ubiquitination.^[^ ^37^ ^]^ Ubiquitin contains seven lysine (K) residues, and ubiquitination can result in different biological outcomes depending on the specific linkages that are formed. K48‐ and K11‐linked polyubiquitin chains are associated with the degradation of the target protein by the 26S proteasome,^[^ ^38^ ^]^ whereas K63‐linked polyubiquitin chains play a scaffolding role in the context of signaling, and can serve as signaling molecules in the context of protein‐protein interaction.^[^ ^38^ ^]^ In contrast, K6 and K27 poly‐ubiquitinated proteins are associated with mitochondrial maintenance and DNA damage responses, respectively.^[^ ^39^ ^]^ As a post‐translational modification, growing evidence has shown that ubiquitination is highly associated with TAM resistance through various mechanisms in human breast cancer. Several E3 ligases such as TRIM8, COP1, BRCA1, RNF31, CHIP, and MDM2 can directly interact with the ER‐*α* and regulate its degradation and TAM sensitivity.^[^ ^40^ ^]^ Irrespective of ER‐*α* activity levels, endocrine resistance is often caused by the deregulation of several key signaling pathways and protein activities. For example, TRIM47, TRIM27, UBR5, and FBXW2 can regulate breast cancer progression and endocrine therapy sensitivities via the polyubiquitination of PKC‐*ε*, P21, *β*‐catenin, and MSX2, respectively.^[^ ^41^ ^]^

SET is a multifunctional protein that regulates cell motility,^[^ ^42^ ^]^ proliferation,^[^ ^43^ ^]^ cell cycle progression,^[^ ^44^ ^]^ and gene transcription by binding to gene promoters.^[^ ^45^ ^]^ SET is expressed in many organs, including the liver, kidney, spleen, lungs, heart, gonadal system, and brain.^[^ ^46^ ^]^ Many studies have shown that SET overexpression plays important role in the development of Alzheimer's disease,^[^ ^47^ ^]^ myeloid leukemia,^[^ ^48^ ^]^ B‐cell chronic lymphocytic leukemia, and non‐Hodgkin lymphoma.^[^ ^49^ ^]^ In this study, we found that TRIM4 enhanced the K48‐linked ubiquitination of SET, but had no effect on its K6, K11, K27, K29, K23, or K63 ubiquitination. Moreover, the enzymatically inactive mutant TRIM4 (C27S) lost the ability to ubiquitinate SET. We also determined that the SET K150 and K172 residues were essential for its TRIM4‐mediated ubiquitination. Overall, our results outline a novel regulatory mechanism governing the ubiquitination and degradation of SET and strongly suggest that the TRIM4‐mediated control of SET expression represents a promising target for the development of novel therapeutic strategies for multiple diseases.

In summary, we herein uncovered a novel mechanism regulating the control of SET and ER‐*α* expression related to TAM resistance. As growing evidence implicates aberrant SET expression in a range of diseases, the modulation of SET expression may be amenable to therapeutic targeting. Specifically, our findings highlight a new approach to regulating SET expression via TRIM4‐induced K48‐linked polyubiquitination and proteasomal degradation of SET, thereby outlining a promising therapeutic target for the remediation of TAM resistance in ER‐*α*‐positive breast cancer and other diseases associated with aberrant SET expression.

## 4 Experimental Section

### Cell Culture

MDA‐MB‐468 (RRID: CVCL_0419), T47D (RRID: CVCL_0553), MCF7 (RRID: CVCL_0031), MDA‐MB‐231 (RRID: CVCL_0062), Hs578T (CVCL_0332) and HEK293T (RRID: CVCL_0063) cells were purchased from American Type Culture Collection (ATCC) (VA, USA) and authenticated based on provided directions. High‐glucose DMEM (HyClone, UT, USA) containing 10% FBS (Gibco, MI, USA) and 1% penicillin/streptomycin (HyClone) was used to culture all cells in a humidified 37 °C 5% CO_2_ incubator. MCF7/TR cells were established as in prior reports and were cultured in media containing TAM (10 µM).^[^ ^50^ ^]^ T47D/TR cells were generated by growing TAM‐sensitive T47D cells in media containing TAM (10 µM TAM) for > 12 months.

### Reagents and Antibodies

TAM (Cat# T5648) (5 µM final concentration for MCF7 and T47D, 10 µM final concentration for MCF7/TR and T47D/TR), MG132 (Cat# 474790) (10 µM final concentration), and *β*‐Estradiol (Cat# E2758) (10 nM final concentration for luminal cells or 20 nM for TNBC cells) were purchased from Sigma‐Aldrich (MO, USA). Chloroquine (Cat# NSC‐187208) (100 µM final concentration), 3‐Methyladenine (3‐MA) (Cat# 5142‐23‐4) (5 mM final concentration), and Cycloheximide (CHX) (Cat# NSC‐185) (10 µM final concentration) were from Selleck (TX, USA). Antibodies used in this study were as follows: anti‐TRIM4 (Cat# ab171613), anti‐Ubiquitin (K48) (Cat# ab140601), anti‐PP2A (Cat# ab32104), anti‐Ubiquitin (K63) (Cat# ab179434) were from Abcam (MA, USA). Anti‐Flag (Cat# F2555) and anti‐HA (Cat# H6908) were from Sigma‐Aldrich. Anti‐*β*‐actin (Cat# 66009‐I‐Ig) and anti‐Ki‐67 (Cat# 27309‐1‐AP) were from Proteintech (IL, USA). Anti‐Myc (Cat# A190‐105A) was from Bethyl Laboratories (TX, USA). Anti‐AKT (Cat# 4691), anti‐p‐AKT (Cat# 4060), anti‐JNK (Cat# 9252), anti‐p‐JNK (Cat# 9251), anti‐ERK (Cat# 4695), anti‐p‐ERK (Cat# 4370), anti‐p38 (Cat# 8690, RRID: AB_10999090), anti‐p‐p38 (Cat# 4511), anti‐ER‐*α* (Cat# 8644) were from Cell Signaling Technology (MA, USA). Anti‐SET (Cat# sc‐133138), anti‐ubiquitin (Cat# sc‐8017), anti‐PR (Cat# sc‐166169) anti‐HER‐2 (Cat# sc‐33684, RRID: AB_2185628), protein A/G agarose (Cat# sc‐2003), anti‐P53(Cat# sc‐126) and HRP‐linked secondary antibodies were purchased from Santa Cruz Biotechnology (Santa Cruz, CA, USA).

### Plasmid Transfection

The MCF7 cell TRIM4 cDNA sequence was amplified via PCR and cloned into the pCDNA3.1‐Flag (RRID: Addgene_42596) and pEGFP‐C1 vectors. The H3.3C and SET cDNA constructs were obtained from Vigene Biosciences and subcloned into the pCMV‐C‐Myc vector. Mutated SET and TRIM4 sequences were prepared with a KOD‐Plus‐Mutagenesis kit (Toyobo, Osaka, Japan), with the primer sequences used for this process being listed in the key resources table. DNA sequencing was performed to confirm the identities of all prepared constructs. TRIM4‐specific shRNA plasmids were obtained from GenePharma (Shanghai, China), with sequences being listed in the key resources table. The pGMLV‐CMV‐H_TRIM4‐3×Flag‐PGK‐Puro lentiviral plasmid was obtained from Genomeditech (Shanghai, China). The sh‐SET‐PTSB‐U6‐PGK‐Fluor‐2A‐ARGs lentiviral plasmid, si‐p53, and si‐PP2A were obtained from Tsingke Biotechnology (Beijing, China), with sequences being listed in the key resources table. The 3×ERE TATA luc (plasmid #11354) luciferase reporter plasmid was obtained from Addgene (MA, USA). HA‐Ub WT and HA‐Ub mutant (K6/K11/K27/K29/K33/K48/K63/K0/K48R/K63R) plasmids were kindly provided by Dr. W. Zhao (Shandong University, Jinan, China). Lipofectamine 2000 (Invitrogen, CA, USA) was used to transfect cells with these constructs based on provided directions.

### Immunoprecipitation (IP) and Immunoblotting (IB)

At 48 h post‐transfection, cells were lysed using a lysis buffer (Cat# P0013) (Beyotime, Jiangsu, China) containing protease inhibitors (Merck Millipore, MA, USA). Protein‐containing lysates were centrifuged for 15 min at 16000 ×g, and supernatants were then mixed with the protein A/G Plus‐Agarose IP reagent and 1 µg of monoclonal anti‐Flag/Myc or appropriate antibodies for 8 h. An NP‐40‐containing buffer (Beyotime) was then used to wash beads five times, after which proteins were eluted by boiling beads in a 1% SDS sample buffer. For Re‐IP assays, the immunoprecipitates were denatured by boiling them in the IP buffer containing 1% SDS. The elutes were diluted 1:10 with IP buffer and then immunoprecipitation was performed as above. Western blotting was then conducted by separating these precipitated proteins or input lysates by 10% SDS‐PAGE, transferring them to PVDF membranes, blocking these blots for 1 h with 1% BSA, and then probing overnight at 4 °C with appropriate primary antibodies. Blots were then stained for 1 h using secondary HRP‐conjugated antibodies, after which protein bands were detected with an ECL kit (Merck Millipore).

### Immunohistochemistry (IHC)

An IHC reagent kit (ZSGB‐BIO, Beijing, China) was used for the immunostaining of 4 µm‐thick formalin‐fixed, paraffin‐embedded sections of tumor tissue. Xylene and an ethanol gradient were used to deparaffinize and dehydrate samples, after which 3% hydrogen peroxide was used for the blocking of endogenous peroxidase activity and non‐specific antigen binding. Antigen retrieval was performed by heating sections in sodium citrate buffer (pH = 6.0) for 30 min at 100 °C in a microwave oven. Sections were then stained overnight at 4 °C with appropriate primary antibodies, rinsed with PBS, stained using Diaminobenzidine (DAB) (ZSGB‐BIO), counterstained using hematoxylin, mounted with neutral gum, and evaluated via light microscopy. Stained IHC sections were then assessed and imaged as in a prior report.^[^ ^51^ ^]^

### Cell Viability and Cytotoxicity Assays

After transfection, cells were added to 96‐well plates (1 × 10^3^ cells well^−1^). Media was then replaced with growth media supplemented with a range of TAM concentrations for 48 h or for a range of durations. At appropriate time points, cells were rinsed using PBS, MTT (Sigma) was added to each well (1 mg mL^−1^) and cells were incubated for an additional 4 h at 37 ˚C. Media was then discarded, and DMSO was used to dissolve formazan crystals. Absorbance at 490 nm was then quantified with a microplate reader (PerkinElmer, Inc, USA). IC_50_ values were determined using GraphPad.

### Flow Cytometry

At 24 h post‐transfection, cells were treated for an additional 24 h with TAM or vehicle control (100% ethanol), after which cells were collected, rinsed with chilled PBS, and stained for 30 min with propidium iodide (PI, Sigma) while protected from light. A flow cytometer (Becton Dickinson, NJ, USA) was then used to assess cell cycle distributions. Data were analyzed using the FlowJo application (v10.6.2) (FlowJo, RRID: SCR_0 08520). Stem cell surface marker expression was assessed at 48 h post‐transfection by staining cells for 15 min with FITC‐anti‐CD44 and PE‐anti‐CD24 (BD Biosciences, CA, USA) at room temperature. FlowJo was then used to establish the frequency of CD44^+^/CD24^−^ cells.

### Luciferase Reporter Assay

A dual‐luciferase reporter assay kit (Promega, WI, USA) was used to conduct reporter assays as in prior reports,^[^ ^27^, ^31^ ^]^ using the pGL3‐(ERE)_3_ and the PRL firefly and Renilla luciferase reporter plasmids, respectively.

### Actinomycin D Assay

Cells were exposed to 2 mg ml^−1^ actinomycin D (Sigma) to inhibit transcription, and *ESR1* mRNA stability was assessed via qRT‐PCR.

### Patient Samples

Tissue samples were collected from breast cancer patients that had undergone histological diagnosis and surgical treatment at Qilu Hospital of Shandong University (Shandong, China) between January and December 2012. Follow‐up data were available for all patients through December 30, 2019. All patients provided written informed consent for study participation. The Ethics Committee on Scientific Research of Shandong University Qilu Hospital approved this study (KYLL‐2016‐255). which was conducted in a manner consistent with the Declaration of Helsinki.

### Orthotopic Xenograft Modeling

Female 6‐week‐old nu/nu BALB/c mice (Charles River Company, Beijing, China) were subcutaneously implanted with (MCF7 group) or without (MCF7/TR group) E2 pellets (0.72 mg pellet^−1^; 60‐day release) 1 week prior to the implantation of cancer cells. Mice were then implanted subcutaneously with 5 × 10^6^ MCF7 cells stably expressing sh‐TRIM4 or sh‐NC or with 5 × 10^6^ MCF7/TR cells stably expressing TRIM4‐Flag or control constructs. Beginning 1‐week post‐implantation, mice were intragastrically administered TAM (5 mg kg^−1^) or control treatment every other day. At the end of the 4‐week study period, mice were euthanized under anesthetization. Tumors were then weighed (mg) prior to collection for IHC analyses of consecutive tumor sections from three xenograft model mice. IHC staining results were scored by multiplying the percentage of positive cells by the staining intensity as detailed in the IHC staining section above. The selected total threshold score was 4 for IHC staining results. Shandong University Animal Care and Use Committee approved all animal studies described herein(KYLL‐2020(KS)‐215).

### Statistical Analysis

Experiments were repeated a minimum of three times. GraphPad Prism 8.4.2 (GraphPad Prism, RRID: SCR_002798) and the SPSS 25.0 (IBM SPSS, RRID: SCR_002865) were used for all statistical analyses. Differences between groups were compared using two‐tailed Student's *t*‐tests. Differences in patient survival outcomes were compared using Kaplan–Meier curves and log‐rank tests. Univariate and multivariate Cox proportional hazard regression models were used to identify independent predictors of patient prognosis. ANOVAs with pairwise comparisons were used to compare differences in TRIM4 expression between different breast cancer subtypes. Correlations were analyzed via Spearman's rank correlation analyses. Data are given as means with the standard error of the mean (SEM) from three experiments. *p* < 0.05 was the threshold of significance in this study.

### Ethics Approval and Consent to Participate

This project was approved by the Ethical Committee on Scientific Research of Shandong University Qilu Hospital.

## Conflict of Interest

The authors declare no conflict of interest.

## Author Contributions

D.H. and L.W. are the co‐first authors. Q.Y. and G.H. designed research studies; L.W., D.H., L.L., P.S, D.L, and H.Z. conducted experiments; Z.L. performed the bioinformatical analysis; B.C., W.Z., N.Z., X.W., Y.L. and Y.L. provided valuable discussion; D.H. acquired data; W.Z. analyzed data; Q.Y. provided reagents; L.W. and D.H. wrote the manuscript.

## Supporting information

Supporting information

## Data Availability

The datasets used and/or analyzed during the current study are available from the corresponding author on reasonable request.
