# Supplementary material for: The E3 Ligase TRIM4 Facilitates SET Ubiquitin‐Mediated Degradation to Enhance ER‐α Action in Breast Cancer
Source: Adv Sci (Weinh). 2022 Jul 17;9(25):2201701. doi: 10.1002/advs.202201701 (PMC9443474; doi:10.1002/advs.202201701)
Supplement: Supplementary file 1 — Supporting information [file ADVS-9-2201701-s001.pdf]

ADVANCED SCIENCE
Open Access

Supporting Information

for *Adv. Sci.*, DOI 10.1002/advs.202201701

The E3 Ligase TRIM4 Facilitates SET Ubiquitin-Mediated Degradation to Enhance ER-α Action in Breast Cancer

*Dianwen Han, Lijuan Wang, Li Long, Peng Su, Dan Luo, Hanwen Zhang, Zheng Li, Bing Chen, Wenjing Zhao, Ning Zhang, Xiaolong Wang, Yiran Liang, Yaming Li, Guohong Hu and Qifeng Yang**

Supplementary Materials for

The E3 ligase TRIM4 facilitates SET ubiquitin-mediated degradation to enhance ER-α action in breast cancer

Dianwen Han[1, †], Lijuan Wang[2, †], Li Long[1, 3], Peng Su[4], Dan Luo[1], Hanwen Zhang[1], Zheng Li[1], Bing Chen[2], Wenjing Zhao[2], Ning Zhang[1], Xiaolong Wang[1], Yiran Liang[1], Yaming Li[1], Guohong Hu[5] and Qifeng Yang[1, 2, 6, *]

[*]**Address correspondence to:** Qifeng Yang (qifengy_sdu@163.com).

Supplementary Text

Materials and methods

qPCR

Trizol (Invitrogen) was used to extract total RNA from cells based on provided directions, after which reverse transcriptase (Takara, Shiga, Japan) was used to prepare cDNA, and qPCR analyses were performed with a SYBR Green RT-PCR kit (Roche, Basel, Switzerland). GAPDH expression was used to normalize gene expression. All qPCR primers used in this study are listed in Supplementary Table 1.

Mass spectrometry analyses.

For mass spectrometry analysis, IP was performed with the whole cell lysates in MCF7 cells transfected with Flag-tagged TRIM4 or control plasmids. The IP proteins were resolved by SDS-PAGE, followed by Coomassie staining and the next mass spectrometry analyses were performed by Jingjie PTM BioLab (Hangzhou, China).

Immunofluorescent (IF) and confocal microscopy

IF analyses were performed as in a prior report [1]. Briefly, MCF7 cells were added onto glass coverslips in 24-well plates. Following TRIM4-GFP and SET-Myc plasmid transfection, cells were incubated for 90 min with anti-Myc without permeabilization. They were then washed two times with PBS, probed with a secondary Alexa Fluor 633-conjugated antibody for 30 min and imaged with a laser-scanning confocal microscope (Nikon confocal microscopy, Eclipse Ti A1).

Colony formation assay.

Colony forming ability were assessed using a colony formation assay which was performed as previously described [2]. The transfected cells were seeded into 6 cm plates at a density of 5×10^3 cells/well for 24 h, followed by treatment with indicated TAM for 48 h. After cultured with new

medium for 2 weeks, the cells were then washed twice with cold PBS, fixed with methanol-glacial acetic acid stationary solution (3:1), and stained with 0.5% crystal violet solution. Pictures were imaged and counted.

Transwell.

Transwell assay was carried out as previously described [3]. Briefly, after transfection, cells underwent a 6 h serum starvation step, 1×10^5 MCF7/TR or T47D/TR cells and 2×10^5 MCF-7 or T47D cells were suspended in 200μL serum-free medium and seeded into the inside of each transwell insert (Corning Costar, USA), while 700 μL medium containing 20% FBS was placed in the lower well. After incubation for 24-72 h, the infiltrating cells on the lower surface were fixed with methanol and stained with 0.1% crystal violet. Cell invasion assay was conducted using the same procedure as in the cell migration assay, except that the inside of each insert was coated with Matrigel (BD Biosciences, San Jose, CA, USA).

Patient-derived organoid (PDO) culture

After mincing, ER-α -positive breast cancer tissue was digested for 3 h using digest medium (10 mM HEPES, 2% BSA, 50 μg/mL gentamycin, 0.5 μg/mL hydrocortisone, 2 mg/mL collagenase IV (Sigma C5138), 1000 U/mL hyaluronidase (Sigma H3884), 1% penicillin/streptomycin and 10% FBS in DMEM) at 37 °C. Cells were then rinsed and suspended in 3D Matrigel with human complete organoid medium composed of advanced DMEM/F12 (Gibco 12634010) containing 10 mM HEPES (Invitrogen 15630-056), 1% penicillin/streptomycin, 1% GlutaMAX (Invitrogen 35050), 50 ng/mL human EGF (PeproTech 315-09, NJ, USA), 1 mM N-acetyl-L-cysteine (Sigma A9165), 100 ng/mL Noggin (Peprotech 250-38), 500 ng/mL R-spondin (BioLegend 783606, CA, USA), 1×N2 (Gibco 17502-048), 1×B27 (Gibco 17504-044), 10 mM Nicotinamide (Sigma N0636), 500 nM A83-01 (STEMCELL Technologies 72024, MA, USA), 10 μM SB202190

(Sigma S7067), 5 μM Y-27632 (Sigma 129830-38-2), 0.5 nM prostaglandin E2 (Selleck S3003), and 1×Primocin (InvivoGen ant-pm-1, CA, USA). Organoids were propagated by dissociating them using TrypLE (Gibco 12604021) prior to re-seeding in fresh Matrigel and culture medium. At 48 h post-seeding, TRIM4-overexpressing or control retroviruses were used to infect these cells. Puromycin (0.5 mg/mL; Sigma) was then used to select cells for 3 weeks, after which the organoids were imaged and counted via microscopy (20×; Zeiss ObserverZ1). Organoids were then treated for an additional 4 days with TAM (15 μM) and imaged at 20×magnification.

Molecular modelling and protein-protein docking.

The amino-acid sequences of TRIM4 (NP_148977.2) and SET (NP_001116293.1) retrieved from the NCBI database were used as targets for homology modeling using I-TASSER (https://zhanglab.ccmb.med.umich.edu/I-TASSER/), and the ZDOCK (version 3.0.2) algorithm with the default parameters was used to predict the structures of the TRIM4-SET complex. The docking results were visualized by visual molecular dynamics (VMD). Discovery studio (version 4.5) was used to build the TRIM4 Cys27 models.

References

[1] M. Jia, D. Qin, C. Zhao, L. Chai, Z. Yu, W. Wang, L. Tong, L. Lv, Y. Wang, J. Rehwinkel, J. Yu, W. Zhao, *Nature immunology* **2020**, *21* (7), 727, https://doi.org/10.1038/s41590-020-0699-0.
[2] L. Xiang, B. He, Q. Liu, D. Hu, W. Liao, R. Li, X. Peng, Q. Wang, G. Zhao, *Oncology reports* **2020**, *44* (5), 1997, https://doi.org/10.3892/or.2020.7765.
[3] C. Wei, X. Zhao, L. Wang, H. Zhang, *FEBS letters* **2020**, *594* (19), 3170, https://doi.org/10.1002/1873-3468.13882.

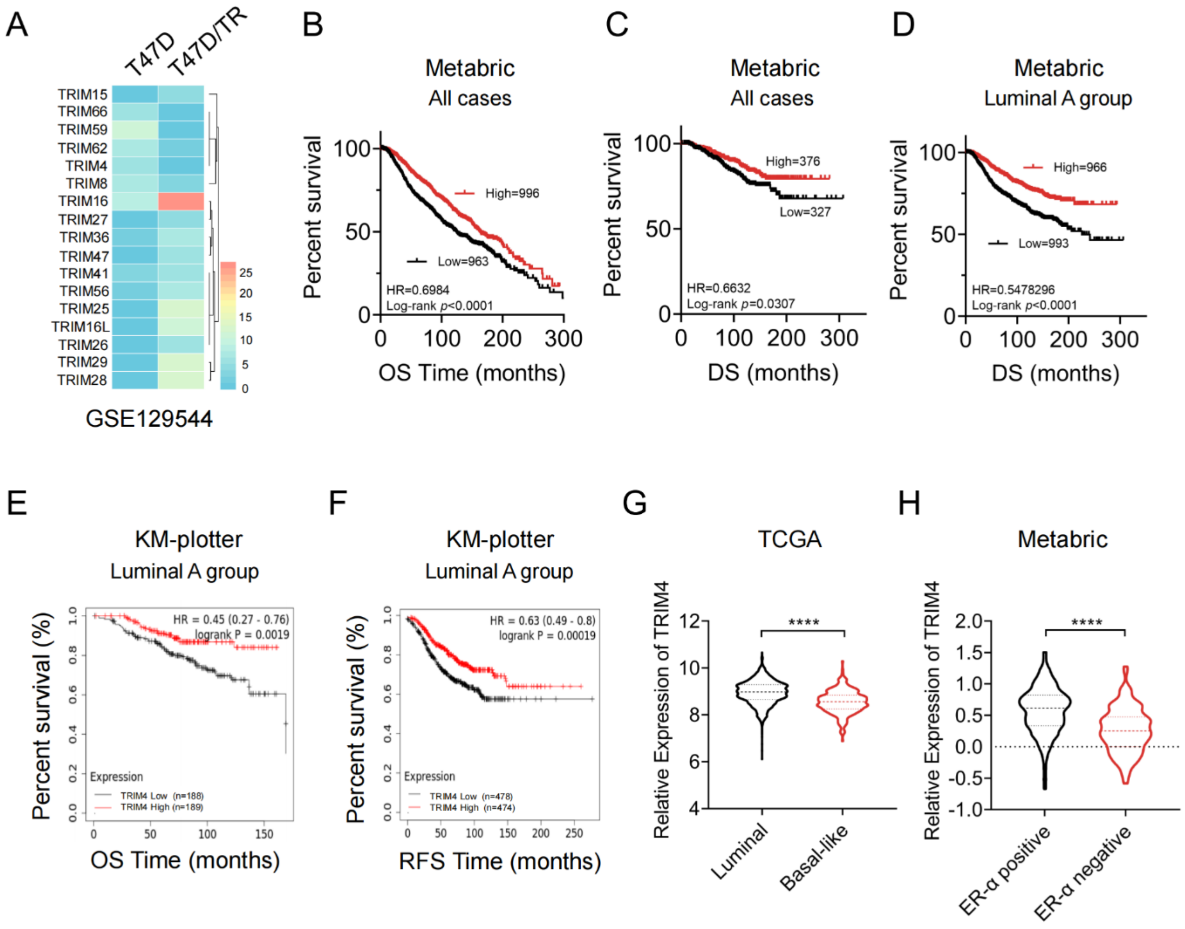

Supplementary Figure 1. Reduced TRIM4 expression is associated with TAM resistance and poor prognosis in breast cancer. (A) GSE129544 data-based heatmap displaying the deregulated genes between T47D and T47D/TR cells. **(B-C)** Overall breast cancer patient OS (B) and DS (C) as a function of TRIM4 expression levels were assessed with the Metabric database. **(D-F)** Luminal A breast cancer patient DS (D), OS (E) and RFS (F) as a function of TRIM4 expression levels were assessed with Metabric and KM-plotter database. **(G)** Correlations between TRIM4 expression and pathological type in breast cancer cohorts (Luminal: n=1661, Basal-like: n=319). **(H)** Correlations between TRIM4 expression and ER-α status in breast cancer cohorts (ER-α

positive: n=398, ER-α negative: n=117). Data information: data were presented as mean ± SD. *Unpaired two-tailed Student's t-test*; ****$p<0.0001$.

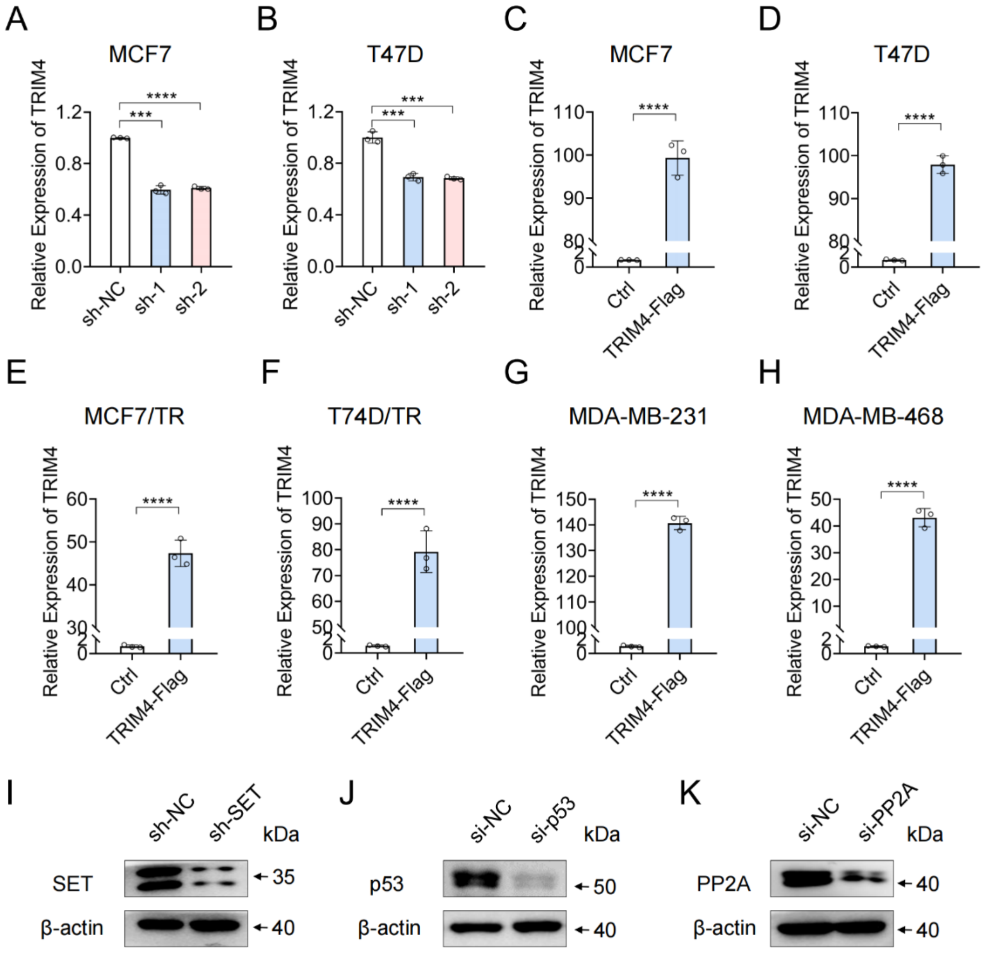

Supplementary Figure 2. TRIM4 knockdown and overexpression efficiencies. (A-B) Efficient knockdown of TRIM4 expression in the indicated cells, as assessed via qPCR (A, B). **(C-H)** Efficient overexpression of TRIM4 expression in the indicated cells, as assessed via qPCR. **(I)** Knockdown efficiency of SET in MCF7 cells. **(J)** Knockdown efficiency of p53 in MCF7 cells. **(K)** Knockdown efficiency of PP2A in MCF7 cells. All data were representative of n=3 independent experiments. Data information: data were presented as mean ± SD. *Unpaired two-tailed Student's t-test*; ***p<0.001; ****p<0.0001.

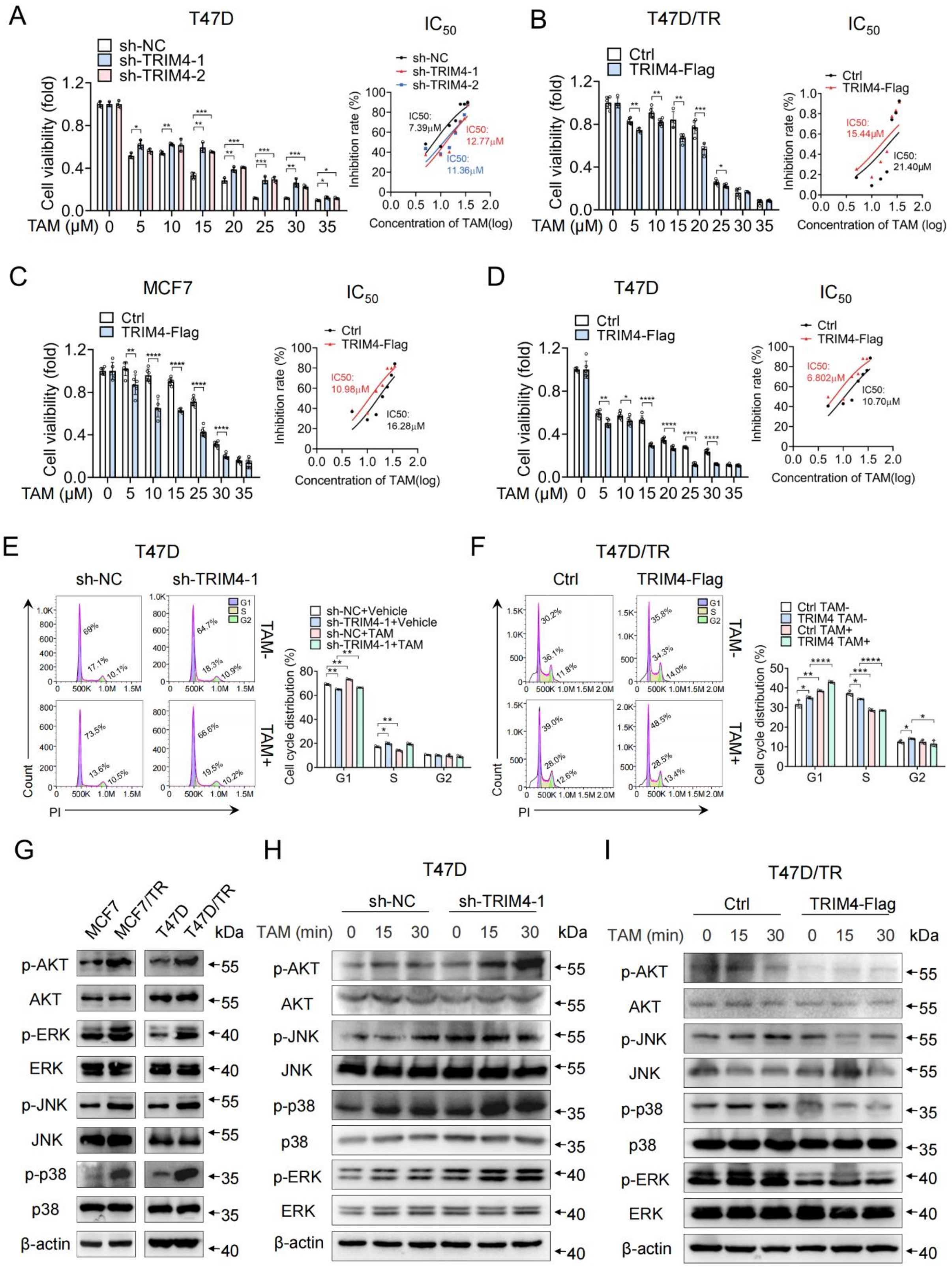
A
T47D
sh-NC
sh-TRIM4-1
sh-TRIM4-2
Cell viability (fold)
TAM (μM) 0 5 10 15 20 25 30 35
IC50
Inhibition rate (%)
IC50: 7.39μM
IC50: 12.77μM
IC50: 11.36μM
Concentration of TAM(log)
B
T47D/TR
Ctrl
TRIM4-Flag
Cell viability (fold)
TAM (μM) 0 5 10 15 20 25 30 35
IC50
Inhibition rate (%)
IC50: 15.44μM
IC50: 21.40μM
Concentration of TAM (log)
C
MCF7
Ctrl
TRIM4-Flag
Cell viability (fold)
TAM (μM) 0 5 10 15 25 30 35
IC50
Inhibition rate (%)
IC50: 10.98μM
IC50: 16.28μM
Concentration of TAM(log)
D
T47D
Ctrl
TRIM4-Flag
Cell viability (fold)
TAM (μM) 0 5 10 15 20 25 30 35
IC50
Inhibition rate (%)
IC50: 6.802μM
IC50: 10.70μM
Concentration of TAM(log)
E
T47D
sh-NC
sh-TRIM4-1
TAM-
TAM+
69%
17.1%
10.1%
64.7%
18.3%
10.9%
73.5%
13.6%
10.5%
66.6%
19.5%
10.2%
Count
PI
sh-NC+Vehicle
sh-TRIM4-1+Vehicle
sh-NC+TAM
sh-TRIM4-1+TAM
Cell cycle distribution (%)
G1 S G2
F
T47D/TR
Ctrl
TRIM4-Flag
TAM-
TAM+
30.2%
36.1%
11.8%
35.8%
34.3%
14.0%
39.0%
28.0%
12.6%
48.5%
28.5%
13.4%
Count
PI
Ctrl TAM-
TRIM4 TAM-
Ctrl TAM+
TRIM4 TAM+
Cell cycle distribution (%)
G1 S G2
G
MCF7 MCF7/TR T47D T47D/TR kDa
p-AKT 55
AKT 55
p-ERK 40
ERK 40
p-JNK 55
JNK 55
p-p38 35
p38 35
β-actin 40
H
T47D
sh-NC
sh-TRIM4-1
TAM (min) 0 15 30 0 15 30 kDa
p-AKT 55
AKT 55
p-JNK 55
JNK 55
p-p38 35
p38 35
p-ERK 40
ERK 40
β-actin 40
I
T47D/TR
Ctrl
TRIM4-Flag
TAM (min) 0 15 30 0 15 30 kDa
p-AKT 55
AKT 55
p-JNK 55
JNK 55
p-p38 35
p38 35
p-ERK 40
ERK 40
β-actin 40

Supplementary Figure 3. Loss of TRIM4 confers *in vitro* breast cancer cell resistance to TAM. (A-D) MTT assays were used to calculate viability levels and TAM IC_{50} values for TRIM4-silenced or control T47D cells (A) and TRIM4-overexpressing or control T47D/TR (B), MCF7 (C) and T47D cells (D) after treatment for 48 h with the indicated TAM doses (in μM). **(E-F)** Cell cycle progression was assessed via flow cytometry in TRIM4-silenced or control T47D cells (E) and in TRIM4-overexpressing or control T47D/TR cells (F) after treatment with TAM for 48 h. **(G)** TAM-resistance related changes in the MAPK and AKT signaling pathways were assessed in TAM-resistant cells and their parental cells via Western blotting. **(H-I)** TAM-resistance related changes in the MAPK and AKT signaling pathways were assessed in TRIM4-silenced or control T47D cells (H) and in TRIM4-overexpressing or control T47D/TR cells (I) via Western blotting. All data were representative of at least 3 independent experiments. Data information: data were presented as mean ± SD. *Unpaired two-tailed Student's t-test*; *p<0.05; **p<0.01; ***p<0.001; ****p<0.0001.

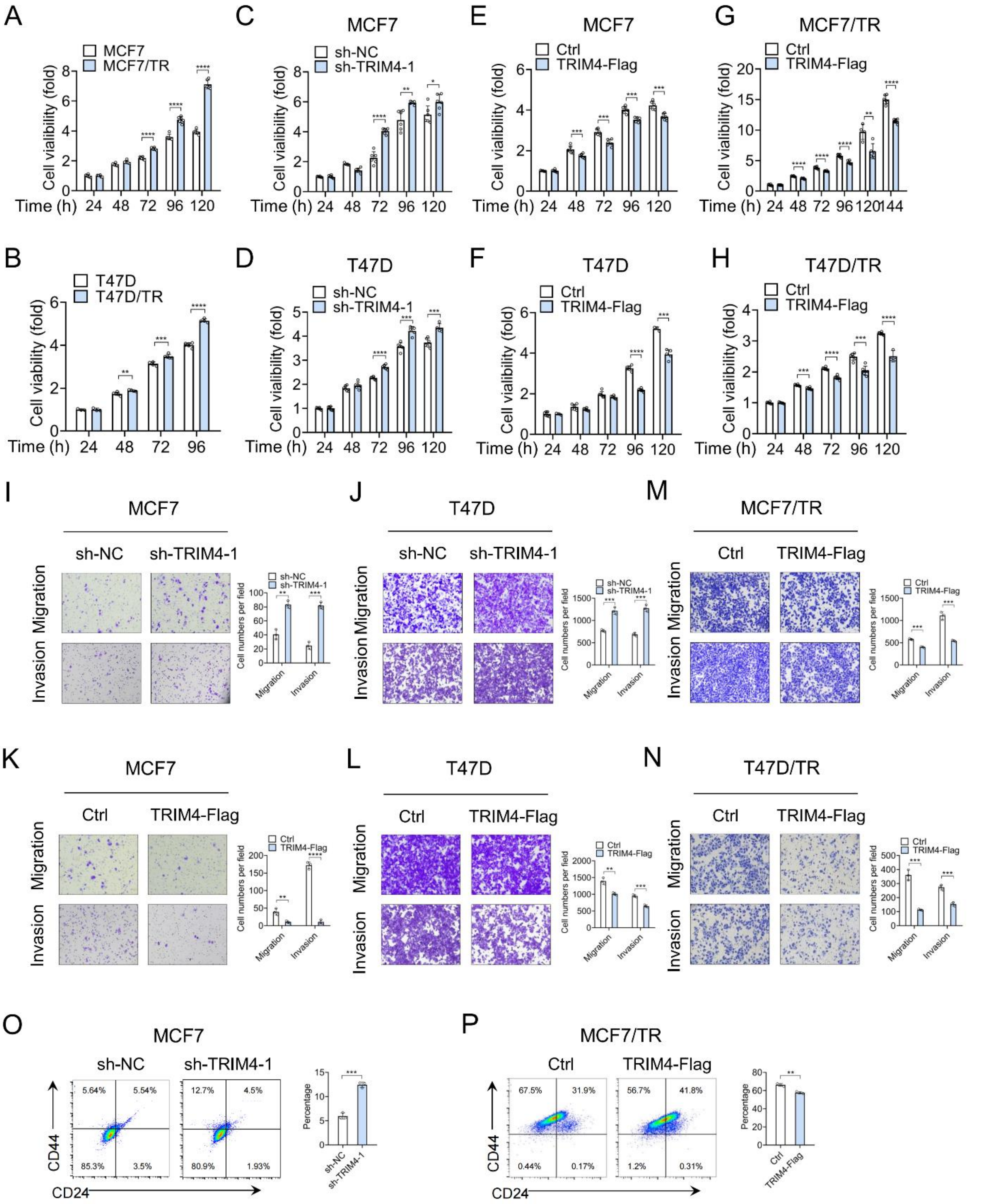

Supplementary Figure 4. TRIM4 attenuates proliferation, metastasis and cell stemness properties of breast cancer cells. (A-B) Cell viabilities of TAM-resistant cells and their parental

cells, as assessed via MTT assay. **(C-D)** Cell viabilities of TRIM4-silenced or control MCF7 (C) and T47D (D) cells. **(E-H)** Cell viabilities of TRIM4-overexpressing or control MCF7 (E), T47D (F), MCF7/TR (G) and T47D/TR (H) cells. **(I-J)** Metastasis capacity of TRIM4-silenced or control MCF7 (I) and T47D (J) cells, as assessed via Transwell assay. **(K-N)** Metastasis capacity of TRIM4-overexpressing or control MCF7 (K), T47D (L), MCF7/TR (M) and T47D/TR (N) cells. **(O-P)** $CD44^{+}CD24^{-}$ cell populations in the prepared TRIM4-silenced MCF7 (O) and TRIM4-overexpressing MCF7/TR (P) cell populations were assessed via flow cytometry. All data were representative of at least 3 independent experiments. Data information: data were presented as mean ± SD. *Unpaired two-tailed Student's t-test*; *p<0.05; **p<0.01; ***p<0.001; ****p<0.0001.

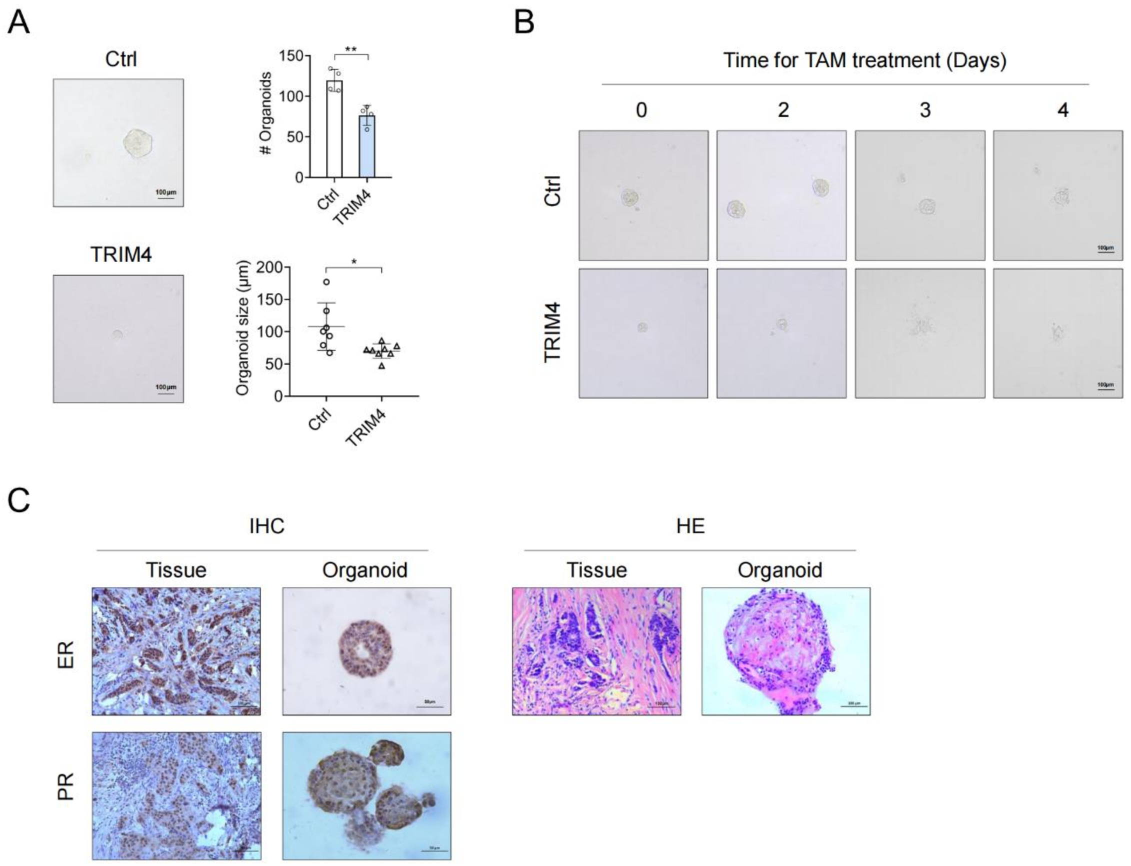

Supplementary Figure 5. Loss of TRIM4 is associated with TAM resistance *in vivo.* (A) Patient-derived breast tumor organoid formation following the overexpression of TRIM4 (n=4 replicate cultures). Data are medians with standard deviations. **(B)** TAM was used to treat TRIM4-overexpressing organoids for the indicated amount of time, with representative images being shown. Scale bars: 100μm. **(C)** Comparative histological and immunohistochemical images of breast cancer and derived organoid lines. Shown are representative examples of ductal carcinoma samples. Tissues generally exhibit a tumor epithelium layer surrounded by mesenchymal and inflammatory cells, whereas organoids are exclusively epithelial, with tumor cell organization being remarkably well conserved (HE). The ER and PR status of these organoid lines was similar to that of the original breast cancers from which they were derived. Scale bar,100 μm. Data

information: data were presented as mean ± SD. *Unpaired two-tailed Student's t-test*; *$p<0.05$; **$p<0.01$.

A

Dataset	n	ESR1	PGR	FOXA1	GATA3
METABRIC(Nature 2012 & Nat Commun 2016)	2509	0.360(<0.001)	0.306(<0.001)	0.414(<0.001)	0.364(<0.001)
Breast Cancer (SMC 2018)	187	0.447(<0.001)	0.483(<0.001)	0.554(<0.001)	0.482(<0.001)
Breast Invasive Carcinoma (TCGA, Cell 2015)	817	0.383(<0.001)	0.381(<0.001)	0.360(<0.001)	0.359(<0.001)
Breast Invasive Carcinoma (TCGA, Nature 2012)	825	0.490(<0.001)	0.358(<0.001)	0.505(<0.001)	0.460(<0.001)
Breast Invasive Carcinoma (TCGA, PanCancer Atlas)	1084	0.372(<0.001)	0.365(<0.001)	0.375(<0.001)	0.331(<0.001)
GSE19615 GPL570		0.448(<0.001)	0.466(<0.001)	0.358(0.001)	0.460(<0.001)
GSE32646 GPL570		0.393(<0.001)	0.429(<0.001)	0.399(<0.001)	

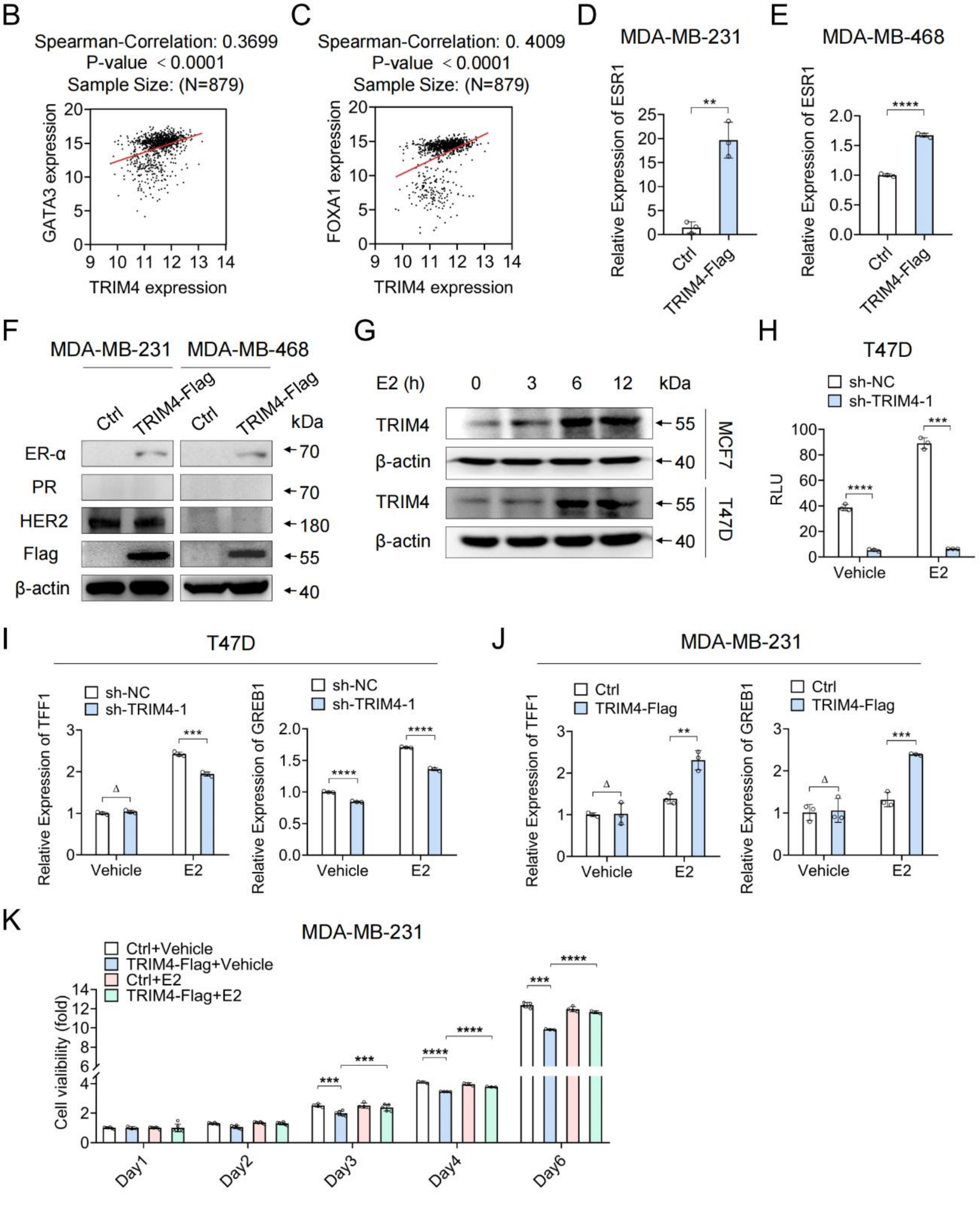

Supplementary Figure 6. TRIM4 induces ER-α upregulation and promotes ER-α-dependent transcriptional activity. (A) The mean expression of the multiple probes for each gene on the Affymetrix GeneChips was used to determine the correlation coefficient. **(B-C)** Scatter plots were used to demonstrate correlations between the expression of TRIM4 and that of *GATA3* and *FOXA1* in the TCGA database. *Spearman rank correlation analyses* were used to establish *r*-values in each group. **(D, E)** The expression of *ESR1* was assessed via qPCR in TRIM4-overexpressing or control MDA-MB-231 (D) and MDA-MB-468 (E) cells. **(F)** The expression of indicated markers was assessed in TRIM4-overexpressing or control indicated cells via Western blotting. **(G)** TRIM4 expression levels were assessed via Western blotting after treatment with E2 for indicated amounts of time. **(H)** ERE-luciferase activity was assessed for TRIM4-silenced or control T47D cells with or without E2. **(I)** *TFF1* and *GREB1* expression levels were assessed via qPCR in TRIM4-silenced or control T47D cells with or without E2. **(J)** *TFF1* and *GREB1* expression levels were assessed via qPCR in TRIM4-overexpressing or control MDA-MB-231 cells with or without E2. **(K)** MTT assay was used to assess the viability of TRIM4-overexpression or control MDA-MB-231 cells treated with the indicated doses of E2 or ethanol (vehicle) for the indicated amount of time. All data were representative of at least 3 independent experiments. Data information: data were presented as mean ± SD. *Unpaired two-tailed Student's t-test*; **p*<0.05; ***p*<0.01; ****p*<0.001; *****p*<0.0001; △, no significance.

A

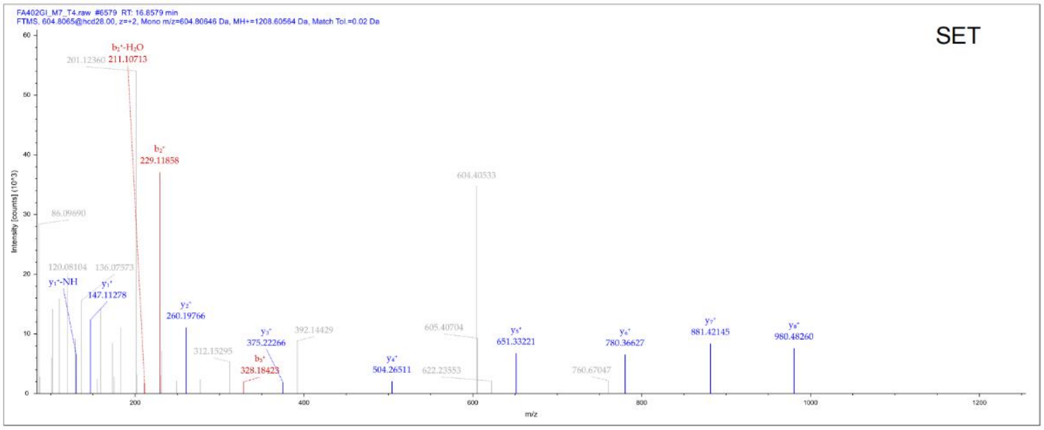

B

	PLMD PTMs: **15**			
	Position	Peptides	Type	References (PMIDs)
Lysine Modification	15	PLPPQKKKRPPPAL	Ubiquitination	26051181
	150	ENPYFENKLSKEFH	Acetylation	19608861; 23236377; 23954790; 25953088; 26051181
	150	ENPYFENKLSKEFH	Sumoylation	27435506
	150	ENPYFENKLSKEFH	Ubiquitination	21139048; 21890473; 21906983; 22053931; 22505724; 23000965;
	154	FENKVLSKFHLNES	Acetylation	23749302; 23954790; 26051181; 26822725; 27452117
	154	FENKVLSKFHLNES	Ubiquitination	18781797; 21139048; 21890473; 21906983; 21963094; 21987572;
	167	ESGDPSSKTEIKWK	Acetylation	23236377; 23954790; 25953088; 26051181; 26210075; 27452117
	167	ESGDPSSKTEIKWK	Malonylation	26320211
	167	ESGDPSSKTEIKWK	Succinylation	23954790
	167	ESGDPSSKTEIKWK	Ubiquitination	21890473; 21906983; 21963094; 23266961; 23503661; 23749302;
	172	SSKSTEIKKSGKDL	Acetylation	19608861; 22424773; 23749302; 25953088; 26051181; 26822725;
	172	SSKSTEIKKSGKDL	Ubiquitination	21890473; 23266961; 23749302; 26051181
	68	QASEEILKEQKYNK	Acetylation	23236377; 26051181; 26822725
	68	QASEEILKEQKYNK	Ubiquitination	21906983; 21963094; 23503661; 24816145; 26051181
	72	EILKVEQKNKLRQP	Acetylation	25953088

C

10 20 30 40 50
MAPKRQSPLP PQKKKPRPPP ALGPEETSAS AGLPKKGEKE QQEAIEHIDE
60 70 80 90 100
VQNEIDRLNE QASEEILKVE QKYNKLRQPF FQKRSELIAK IPNFWVTTFV
110 120 130 140 150
NHPQVSALLG EEDEEALHYL TRVEVTEFED IKSGYRIDFY FDENPYFENK
160 170 180 190 200
VLSKEFHLNE SGDPSSKSTE IKWKSGKDLT KRSSQTQNKA SRKRQHEEPE
210 220 230 240 250
SFFTWFTDHS DAGADELGEV IKDDIWPNPL QYYLVPDMDD EEGEGEEDDD
260 270 280 290
DDEEEEGLED IDEEGDEDEG EEDEDDDEGE EGEEDEGEDD

Supplementary Figure 7. Potential ubiquitination sites in human SET. (A) LC-MS/MS analysis revealing the protein SET precipitated by anti-Flag antibodies from the lysates of MCF7

cells transfected with TRIM4-Flag or control. **(B)** Potential ubiquitination sites were predicted using the Protein Lysine Modification Database (8 lysine (K) residues). **(C)** The position of the predicted ubiquitination sites in the full-length SET amino acid sequence.

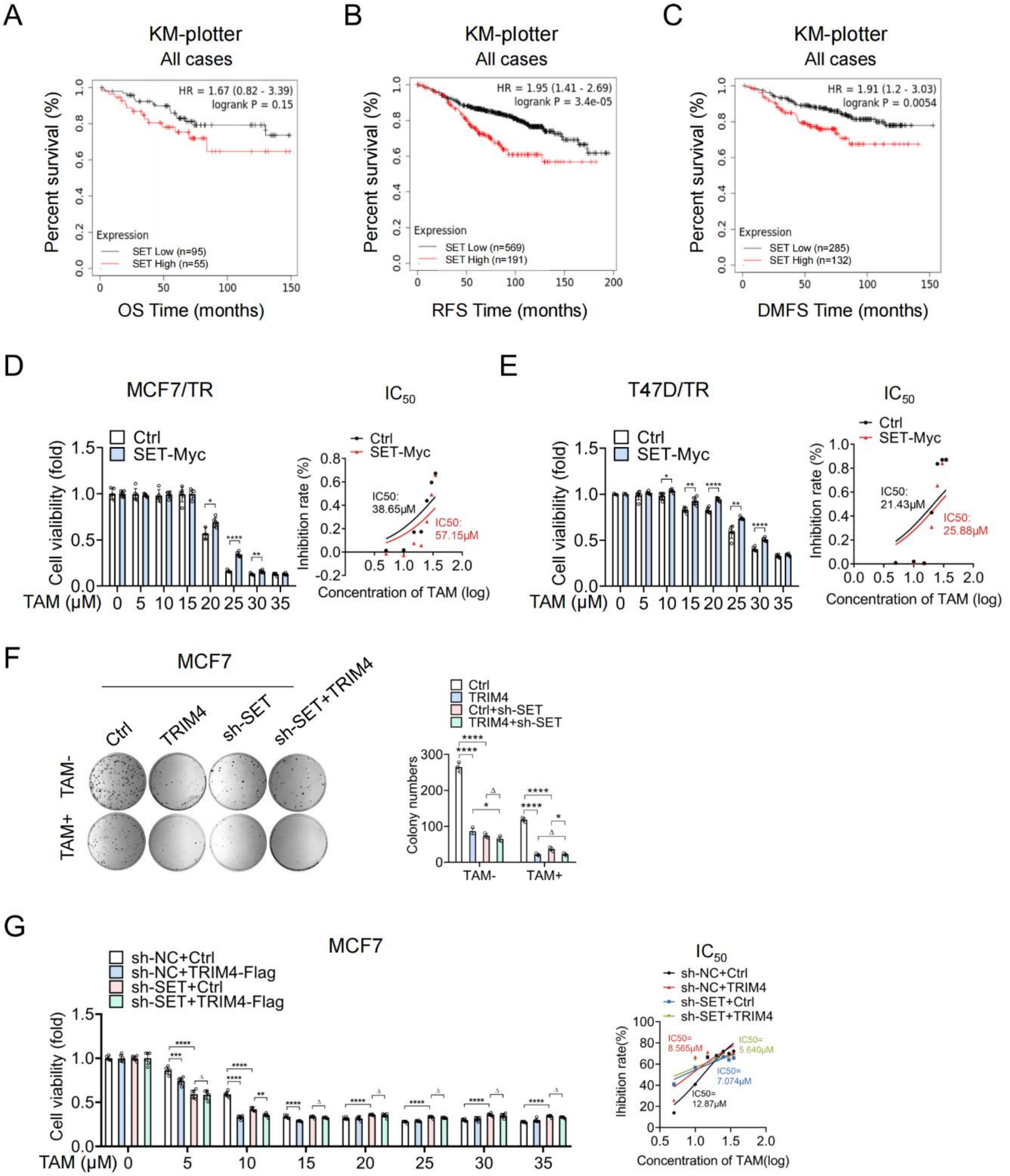

Supplementary Figure 8. SET is associated with poor prognosis and promotes TAM resistance *in vitro*.

(A-C) OS (A), RFS (B), and DMFS (C) in ER-α-positive breast cancer patients following TAM treatment were assessed as a function of SET expression level with the KM-plotter database. **(D-**

E) MTT assays were used to calculate viability levels and TAM IC_{50} values for SET-overexpressing or control MCF7/TR (D) and T47D/TR (E) cells after treatment for 48 h with the indicated TAM doses (in μM). **(F)** Representative images of colonies formed from sh-NC or sh-SET MCF7 cells expressing the indicated plasmids after treatment with TAM (5 μM) for 24h. **(G)** MTT assays were used to calculate viability levels and TAM IC_{50} values for sh-NC or sh-SET MCF7 cells transfected as indicated. All data were representative of at least 3 independent experiments. Data information: data were presented as mean ± SD. *Unpaired two-tailed Student's t-test*; *p<0.05; **p<0.01; ***p<0.001; ****p<0.0001; Δ, no significance.

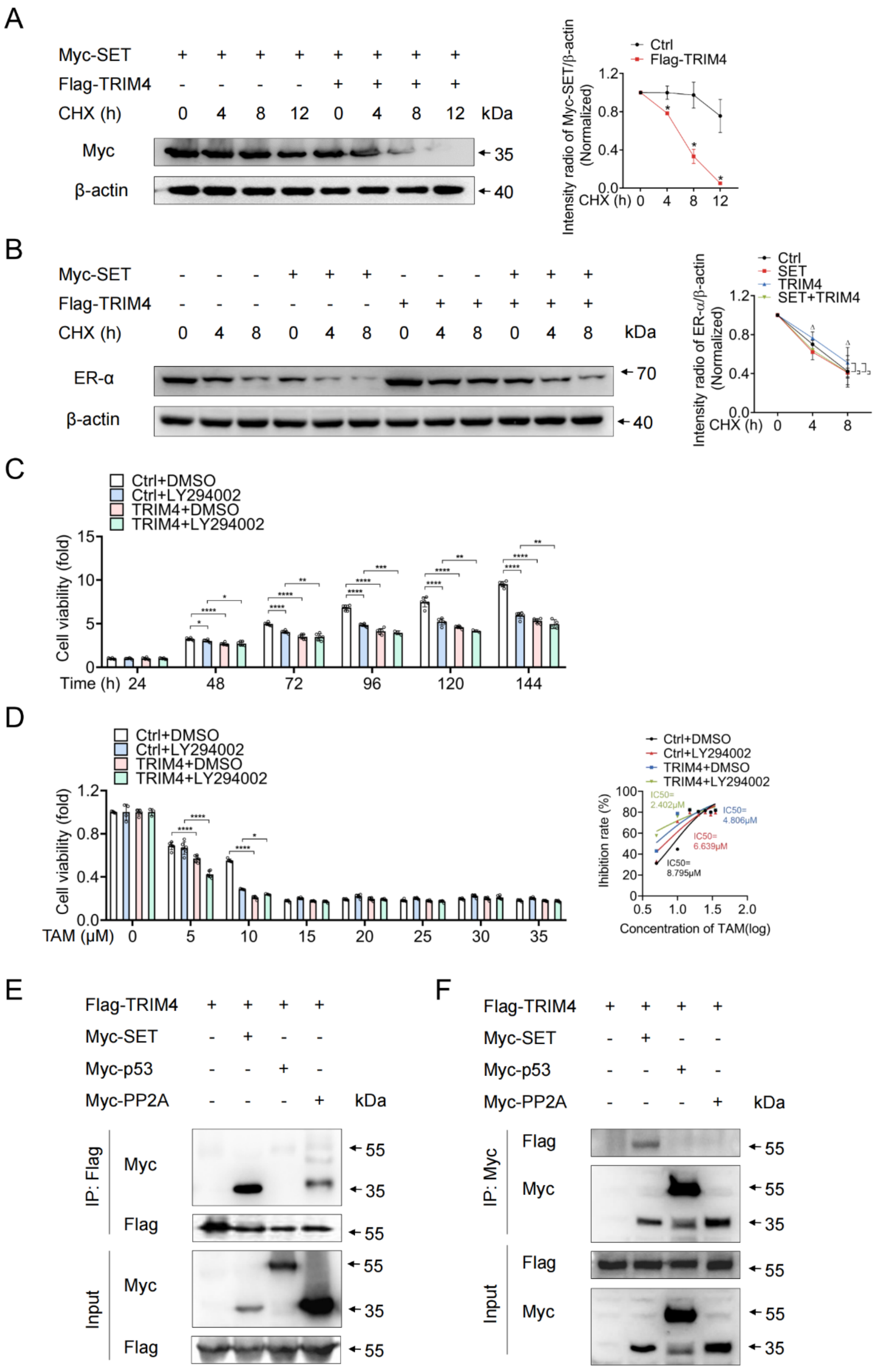

Supplementary Figure 9. PI3K is a major target in the TRIM4-mediated inhibition of

breast cancer proliferation. (A) HEK-293T cells were transfected using the indicated plasmids, followed by CHX treatment for the indicated duration. Immunoblotting was then performed using lysates prepared from these cells, with the ImageJ software being used to quantify Myc-SET expression and β-actin being used for normalization. **(B)** MCF7 cells were transfected using the indicated plasmids, followed by CHX treatment for the indicated duration. Immunoblotting was then performed using lysates prepared from these cells, with the ImageJ software being used to quantify ER-α expression and β-actin being used for normalization. **(C)** MTT assays were used to assess the viability of TRIM4-Flag or control MCF7 cells that had been treated with the indicated doses of LY294002 or ethanol (vehicle) for the indicated amount of time. **(D)** MTT assays were used to calculate viability levels and TAM IC_{50} values for the groups as indicated. **(E-F)** HEK293T cells were transfected with Flag-TRIM4 and the indicated Myc-tagged plasmids, after which IP was performed to assess interactions between TRIM4, SET, p53, and PP2A using anti-Flag (E) or anti-Myc (F) antibodies; IB, immunoblot. All data were representative of at least 3 independent experiments. Data information: data were presented as mean ± SD. *Unpaired two-tailed Student's t-test*; $*p<0.05$; $**p<0.01$; $***p<0.001$; $****p<0.0001$; △, no significance.

Supplementary Table 1. Key resources used in this study

REAGENT or RESOURCE	SOURCE	IDENTIFIER
**Antibodies**		
anti-rabbit TRIM4	Abcam	ab171613
anti-rabbit Ubiquitin (K48-linked)	Abcam	ab140601
anti-rabbit Ubiquitin (K63-linked)	Abcam	ab179434
anti-mouse PR	Santa Cruz Biotechnology	sc-166169
anti-mouse HER-2	Santa Cruz Biotechnology	sc-33684
anti-mouse SET	Santa Cruz Biotechnology	sc-133138
Anti-mouse Ubiquitin	Santa Cruz Biotechnology	sc-8017
anti-rabbit Flag	Sigma-Aldrich	F2555
anti-rabbit HA	Sigma-Aldrich	H6908
anti-mouse β-actin	Proteintech	66009-1-Ig
anti-Ki-67	Proteintech	27309-1-AP
anti-rabbit Myc	Bethyl Laboratories	A190-105A
anti-rabbit AKT	Cell Signaling Technology	#4691
anti-rabbit p-AKT	Cell Signaling Technology	#4060
anti-rabbit JNK	Cell Signaling Technology	#9252
anti-rabbit p-JNK	Cell Signaling Technology	#9251
anti-rabbit ERK	Cell Signaling Technology	#4695
anti-rabbit p-ERK	Cell Signaling Technology	#4370
anti-rabbit p38	Cell Signaling Technology	#8690
anti-rabbit p-p38	Cell Signaling Technology	#4511
anti-rabbit ER-α	Cell Signaling Technology	#8644
anti-mouse IgG	Cell Signaling Technology	#7076
anti-rabbit IgG	Cell Signaling Technology	#7074
Anti-P53	Santa Cruz Biotechnology	sc-126
Anti-PP2A	Abcam	ab32104
**Reagents**		
Tamoxifen	Sigma-Aldrich	T5648
MG132	Sigma-Aldrich	474790
β-Estradiol	Sigma-Aldrich	E2758
Chloroquine	Selleck	NSC-187208
Cycloheximide	Selleck	NSC-185
3-Methyladenine	Selleck	5142-23-4
Ly294002	Selleck	S1105
**Critical Commercial Assays**		
BCA Protein Assay Kit	Thermo Scientific	71285-3
Cell lysis buffer for Western and IP	Beyotime Biotechnology	P0013
KOD-Plus-Mutagenesis kit	Toyobo	SMK-101

Dual-Luciferase Reporter Assay System	Promega	E1960
PrimeScript RT reagent Kit with gDNA	Accurate Biology<br>Takara	AG11706<br>RR047A
DNA SYBR Green I Master kit	Roche	04707516001
**Experimental Models: Cell Lines**		
MCF7 cells	American Type Culture Collection	N/A
T47D cells	American Type Culture Collection	N/A
MDA-MB-231 cells	American Type Culture Collection	N/A
MDA-MB-468 cells	American Type Culture Collection	N/A
HEK293T cells	American Type Culture Collection	N/A
**Experimental Models: Mice**		
BALB/c nu/nu	Charles river company	N/A
**Oligonucleotides**		
TRIM4	F	AAGGTGAAGACAGTGTGCCA
	R	CAGGGAAGCCTATCAAGGGC
TRIM16	F	TGACACCAGAAGAGTGAAGGC
	R	TATTTGCGCTGAACAACGGC
TRIM59	F	TTGTCACCTGCCCTGAAC
	R	TCCTTATCGCCTTGGATC
TRIM66	F	GCCCTCTGTGCTACTTACTC
	R	GCTGGTTGTGGGTTACTCTC
TRIM62	F	TACTGGGAGGTGGTGGTGTC
	R	CGTCGGCGTTGTAGAAGATG
TRIM8	F	CCTATCTGCCTGCACGTTTT
	R	GTTGTAGGCCTGGTTGCACT
TRIM28	F	AGCGGGTGAAGTACACCAAG
	R	ACCCAAAGCCATAGCCTTCC
TRIM25	F	GCCTGGTGGAGCATAAGACC
	R	TTGCAGTCATCCGCACATCC
TRIM29	F	ATGCTTGGTGGTCACTTTGG
	R	GCACTTCCCTTACCAGCATAG
TRIM47	F	GTCCAAAGTCCTGAGCGCC
	R	GCTACGGCTGCACTCTTGAT
TRIM27	F	TTGGGAAGGAATCAGCAGGT
	R	ATCCCTGGAAAGAAGCCTCC
TRIM15	F	TCCCTGAAGGTGGTCCATGAG

	R	CAGGATCTTGCCCGAGGATT
TRIM26	F	AAGTTGTGCGAGCGACAC
	R	CTCAGGTGGTTCAGGATTTT
TRIM36	F	CGTCGGTCCTCCAGAGTTTGTG
	R	GTGGCAAGTTCCGTCGTCTTCC
TRIM56	F	CTGCTTGGMGACTTCCTGAC
	R	GTGGATGGTSCCGTTACTGAG
TRIM41	F	AGCTCTTCTGCGAGGTAGAC
	R	CTCTTCAGCCAGAAACCGTG
TFF1	F	AATAAGGGCTGCTGTTTCG
	R	ACTCCTCTTCTGGAGGGAC
GAPDH	F	GGAGCGAGATCCCTCCAAAAT
	R	GGCTGTTGTCATACTTCTCATGG
PGR	F	GTCAGTGGGCAGATGCTGTA
	R	AGCCCTTCCAAAGGAATTGT
GREB1	F	ATTCCAGCACCCAAAATCTG
	R	GTGCCTCCGTGTCTAGCTTC
SET	F	CATCTTCGAAGTCCACCGAAATC
	R	TGCATCAGAATGGTCAGTAAACCAG
ERα	F	CCTGATGATTGGTCTCGTCTG
	R	GGCACACAAACTCCTCTCC
**Generating mutant plasmid**		
Myc-SET-K15R	F	AGACCAAGACCACCTCCTGCTCTGG
	R	CTTCTTTTGAGGCGGGAGTGGAGAC
Myc-SET-K68R	F	AGAGTAGAACAGAAATATAACAAAC
	R	CAAAATCTCCTCACTGGCTTGTTCA
Myc-SET-K150R	F	AGAGTTCTCTCCAAAGAATTTCATC
	R	ATTTTCAAAGTAAGGATTTTCATCA
Myc-SET-K154R	F	AGAGAATTTCATCTGAATGAGAGTG
	R	GGAGAGAACTTTATTTTCAAAGTAA
Myc-SET-K167R	F	AGGTCCACCGAAATCAAATGGAAAT
	R	CGAAGATGGATCACCACTCTCATTC
Myc-SET-K172R	F	AGATGGAAATCTGGAAAGGATTTGA
	R	GATTTCGGTGGACTTCGAAGATGGA
Myc-SET ΔC	F	CCCAAGCTTATGGCCCCTAAACGCCAGT
	R	CCGCTCGAGTTATTGGCGGAGTTTGTTATATTTCT
Myc-SET ΔN	F	CCCAAGCTTATGCCATTTTTTCAGAAGAGGTC
	R	CCGCTCGAGTTAGTCATCTTCTCCTTCATCCTCC
Flag-TRIM4 C27S	F	AGCGGCCACAACTTCTGCCGCGGCT
	R	CTCGATGGACACCGGGTCCTGGAAA
Flag-TRIM4 ΔRING	F	CACCCATCGGCGCCCGCCG
	R	GGTCAACTCCTCCTGGATGTCCTCA
Flag-TRIM4 ΔB-box	F	GACGAGGCCTTCGAGAGCTA

	R	GGGGCCCAGGCGCCGGCGCT
Flag-TRIM4 ΔCC	F	GGGGAGAAGAGCCAGGCTCCCA
	R	AACCAGGAAGTTGTGCAGCTTTGAA
Flag-TRIM4 ΔSPRY	F	CCAGTGACTGATAGGAAATGA
	R	TACAGCTTCAAGAGAATAGTTCACA
**ShRNA sequence**		
human TRIM4-1		5′-GAAGTTGAGAGTAGAGATA-3′
human TRIM4-2		5’-GAGATTGAACAAAGAAGAA-3′
Sh-SET		5’-CCCGACATGGATGATGAAGAA-3′
Si-P53		5’-GACUCCAGUGGUAAUCUAC-3’
Si-PP2A		5'- GCAAAUCACCAGAUACAAATT-3'

Supplementary Table 2.

Correlation between SET expression and clinical parameters in 116 ER-α positive breast cancer patients

Characteristics	SET-low (n=69)	SET-high (n=47)	*p* value
Age			
<59	53(76.81%)	39(82.98%)	0.4208
>=59	16(23.19%)	8(17.02%)	
Tumor size			
<=T1	49(71.01%)	18(38.30%)	
>T1	14(20.29%)	28(59.57%)	**<0.0001**
Unexamined	6(8.70%)	1(2.13%)	
LN metastasis			
0	51(73.91%)	21(44.68%)	
1-3	14(20.29%)	10(21.28%)	**0.0002**
>3	4(5.80%)	16(34.04%)	
Histologic Grade			
G1	5(7.25%)	4(8.51%)	
G2	42(60.87%)	29(61.70%)	0.2899
G3	6(8.70%)	10(21.28%)	
Unexamined	16(23.18%)	4(8.51%)	
HER-2			
Neg	53(76.80%)	31(65.96%)	0.5023
Pos	6(8.70%)	2(4.26%)	
Unexamined	10(14.50%)	14(29.79%)	
Ki-67			
<=14%	27(39.13%)	11(23.40%)	0.0596
>14%	40(57.97%)	36(76.60%)	
Unexamined	2(2.90)	0(%)	
PR			
Neg	8(11.60%)	5(10.64%)	0.8727
Pos	61(88.40%)	42(89.36%)	

Data are n (%)

All patients were divided into SET low (SI<=4, n=69) and SET high (SI>4, n=47) group, clinical variates included patient age, tumor size, LN metastasis, histological grade and the expression of HER-2 and Ki-67 were divided into categorical data, and the frequency was calculated. *p* values were determined by *two-tailed Chi-square test*.

Supplementary Table 3.

Effect of variates on OS in 116 breast cancer patients in univariate and multivariate Cox proportional hazards regression model

Variable	Univariate analysis		Multivariate analysis	
	HR (95% CI)	*p*	HR (95% CI)	*p*
Age (>=59 vs. <59)	4.241(1.060-16.967)	**0.041**	6.888(1.318-35.990)	**0.022**
Histologic Grade				
G2 vs. G1	25863.077(0.000-2.271E+166)	0.957	-	-
G3 vs. G1	44996.911(0.000-3.958E+166)	0.955	-	-
Unknown vs. G1	17768.976(0.000-1.567E+166)	0.959	-	-
Tumor Size (>T1 vs. <=T1+ Uknown)	0.568(0.115-2.816)	0.489	-	-
LN metastasis				
1-3 vs. 0	1.012(0.105-9.737)	0.992	0.265(0.020-3.538)	0.315
>3 vs. 0	5.196(1.162-23.230)	**0.031**	1.573(0.248-9.978)	0.631
PR Status (pos vs. neg)	0.883(0.109-7.183)	0.908	-	-
HER-2 Status (pos vs. neg + unknown)	1.984(0.243-16.171)	0.522	-	-
Ki-67 Status (pos vs. neg + unknown)	3.881(0.477-31.595)	0.205	-	-
ER-α expression				
++ vs. +	25469.281（0.000-1.244E+178）	0.960		
+++ vs. +	24453.935(0.000-1.192E+178)	0.961		
SET expression	2.513(0.600-10.525)	0.207		
TRIM4 Expression	0.094(0.012-0.761)	**0.027**	0.078(0.007-0.846)	**0.036**

Variates included patient age, tumor size, LN metastasis, histological grade and the expression of ER-α, HER-2, Ki-67, TRIM4 and SET were analyzed in univariate Cox regression model, and the *p* values were determined by univariate Cox regression analysis. For the multiple Cox regression analysis, histological grade, tumor size, HER-2 and Ki-67 status were excluded due to the *p* value larger than 0.05. The variates with a *p* value smaller than 0.05 in univariate analysis were included in the multiple Cox regression model, and the *p* values were determined by multivariate Cox regression analysis, no adjustments were made for multiple comparisons.
